# Prezygotic barriers effectively limit hybridization in a rapid evolutionary radiation

**DOI:** 10.1111/nph.20187

**Published:** 2024-10-14

**Authors:** Kathryn A. Uckele, Oscar M. Vargas, Kathleen M. Kay

**Affiliations:** ^1^ Department of Ecology and Evolutionary Biology University of California Santa Cruz CA 95060 USA; ^2^ Department of Biological Sciences California State Polytechnic University Humboldt, Arcata CA 95521 USA

**Keywords:** *Costus*, evolutionary radiation, hybridization, introgression, phylogenomic methods, prezygotic barriers, speciation, spiral gingers

## Abstract

- Hybridization is increasingly recognized as an important evolutionary process across the tree of life. In many clades, phylogenomic approaches have permitted unparalleled insight into the extent and frequency of hybridization. However, we continue to lack a deep understanding of the factors that limit and shape patterns of hybridization, especially in evolutionary radiations. In this study, we characterized patterns of introgression across *Costus* (Costaceae), a young evolutionary radiation of tropical understory plants that maintain widespread interfertility despite exhibiting strong prezygotic reproductive isolation.
- We analyzed a phylogenomic dataset of 756 genes from 54 *Costus* species using multiple complementary approaches – *D*‐statistics, gene‐tree‐based tests, and phylogenetic network analyses – to detect and characterize introgression events throughout the evolutionary history of the radiation.
- Our results identified a moderate number of introgression events, including a particularly ancient, well‐supported event spanning one of the deepest divergences in the clade. Most introgression events occurred between taxa or ancestral lineages that shared the same pollination syndrome (bee‐pollinated or hummingbird‐pollinated).
- These findings suggest that prezygotic barriers, including pollinator specialization, have been key to the balance between introgression and reproductive isolation in *Costus*.

## Introduction

A growing body of research suggests that hybridization is more prevalent across the tree of life than previously thought, challenging traditional views of speciation as a strictly bifurcating process (Mallet *et al*., 2016; Dagilis *et al*., 2022). Genome‐scale data have illuminated the extent to which ancient hybridization has reshaped the genomes of many extant species (Moran *et al*., 2021), including our own (Sankararaman *et al*., 2016), spurring interest in the evolutionary causes and consequences of hybridization.

Hybridization is common in plants (Stebbins, 1969; Whitney *et al*., 2010) and has shaped genetic variation across numerous plant lineages (Arnold, 1997). However, varying rates of hybridization among different plant clades suggest this process has played a more significant role in some lineages than others (Whitney *et al*., 2010; Barker *et al*., 2016). In angiosperms, hybridization frequently precedes evolutionary innovations and the origin of new lineages. Allopolyploidization, a prevalent mode of hybrid speciation in plants, is implicated in the origin of over 10% of species in a survey of 47 vascular plant genera (Barker *et al*., 2016). By contrast, homoploid hybrid species, which arise without a change in ploidy, are thought to be rarer and require strong extrinsic barriers to occur (Buerkle *et al*., 2000; Abbott *et al*., 2010).

Introgression, another common outcome of hybridization, has been the focus of recent research on adaptation and speciation in plants (Le Corre *et al*., 2020; Todesco *et al*., 2020; Nelson *et al*., 2021). Introgression involves the transfer of genetic material through backcrossing, where hybrids mate with pure individuals. The extent and direction of introgression can inform our understanding of reproductive isolation and adaptation. For example, asymmetric introgression, where backcrossing favors one parental species over the other, can reflect asymmetry in reproductive isolation (Arnold *et al*., 2008). Additionally, the proportion of the genome inherited through interspecific gene flow can shed light on the strength and genetic basis of reproductive barriers (Borge *et al*., 2005; Currat & Excoffier, 2011), though neutral abiotic factors and demographic and genetic processes may also influence the extent and direction of introgression (Currat *et al*., 2008; Bertola *et al*., 2020).

Recent studies increasingly highlight the adaptive role of introgression in transferring beneficial alleles among species (Stankowski & Streisfeld, 2015; Lewis *et al*., 2019; Todesco *et al*., 2020; Baiz *et al*., 2021). Adaptive introgression is believed to underlie the rapid diversification of many adaptive radiations, including Darwin's finches (Grant & Grant, 2019), *Heliconius* butterflies (Pardo‐Diaz *et al*., 2012), and *Rhagoletis* fruit flies (Feder *et al*., 2005). While reproductive barriers are critical to the balance between hybridization and speciation, we are still in the early stages of identifying the specific barriers that influence the frequency and outcome of hybridization over macroevolutionary timescales.

Phylogenomic datasets and coalescent‐based phylogenetic approaches have significantly enhanced our ability to resolve complex evolutionary histories, including rapid and recent evolutionary radiations (Meyer *et al*., 2017; Irisarri *et al*., 2018; Suvorov *et al*., 2022), providing valuable resources for subsequent analyses of introgression. Additionally, genome‐scale datasets and advances in analytical approaches have improved our ability to document fine‐scale patterns of hybrid ancestry across the genomes of diverse species (Sankararaman *et al*., 2016; Schumer *et al*., 2018) and characterize patterns of introgression across entire clades (Morales‐Briones *et al*., 2018a; Suvorov *et al*., 2022). There are numerous approaches that detect introgression within a phylogenetic context, ranging in complexity from ‘test‐based’ approaches that fit a simple phylogenetic tree model to a species quartet (e.g. *D*‐statistic) to parameter‐rich ‘model‐based’ approaches that fit complex historical graph models (e.g. phylogenetic network inference). The *D*‐statistic and related tests (Green *et al*., 2010; Durand *et al*., 2011) are performed on three in‐group taxa or populations at a time, and an outgroup taxon or population is used to polarize ancestral states. To characterize introgression across a phylogeny, tests may be conducted exhaustively across all possible trios of populations or taxa. By contrast, model‐based Bayesian and likelihood methods utilize data from all samples simultaneously to extensively explore phylogenetic network space and jointly estimate the species tree topology and hybridization events. While powerful, network inference is computationally challenging, and current approaches are limited to small datasets. Alternatively, systems of three and four‐sample tests are computationally tractable and generally more robust to common model violations (Hibbins & Hahn, 2022). Still, they can become difficult to interpret when large numbers of correlated tests are significant. In this study, we leverage both model‐based and test‐based methods to detect introgression across the spiral ginger (*Costus*, Costaceae) radiation of Central and South America (hereafter referred to as American *Costus*).

*Costus* represents one of the fastest known plant radiations, with recent estimates suggesting that 78 species have arisen over the last *c*. 3 million years (Myr) (Vargas *et al*., 2020; Maas *et al*., 2023). Recent phylogenetic analyses (Valderrama *et al*., 2020; Vargas *et al*., 2020) have provided well‐supported hypotheses for this clade despite extensive genealogical discordance. However, the extent to which hybridization contributed to observed levels of gene tree conflict is unknown. American *Costus* species are widely interfertile in experimental crosses (Kay & Schemske, 2008) and retain consistent ploidy and high‐genomic synteny across the deepest phylogenetic nodes (Harenčár *et al*., 2023). Whereas *Costus* species are often found in broad geographic sympatry (Vargas *et al*., 2020), hybrid zones are rare in the field (Chen & Schemske, 2015), forming only occasionally in areas of anthropogenic disturbance (Sytsma & Pippen, 1985; Surget‐Groba & Kay, 2013). Hybridization may be curtailed in *Costus* by predominantly prezygotic reproductive isolating barriers, especially ecogeographic and floral isolation, which have been studied extensively in the genus (Kay & Schemske, 2003; Kay, 2006; Chen & Schemske, 2015). Prior research has shown that potential pollen flow among sympatric species is significantly reduced between species with divergent pollination syndromes (bee vs hummingbird) (Kay & Schemske, 2003) and even between morphologically divergent species that share pollinators (Kay, 2006; Chen, 2013). A recent ancestral state reconstruction of pollination syndrome suggests that there have been numerous independent transitions from euglossine bee to hummingbird pollination characterized by evolutionary convergence in key floral traits that increase visitation by hummingbirds or deter bees (Kay & Grossenbacher, 2022).

We used phylogenetic approaches to detect clade‐wide signatures of introgression across 54 American *Costus* taxa using a phylogenomic dataset of 756 genes. Our analyses ranged from simple four‐taxon tests to parameter‐rich model‐based approaches. We detected evidence for a moderate number of introgression events in American *Costus*, including one event that mapped to one of the earliest divergences in the radiation. All but one of these events occurred between taxa or lineages that share the same pollination syndrome (e.g. both bee‐pollinated or both hummingbird‐pollinated), suggesting that pollinator specialization plays an important role in maintaining species boundaries.

## Materials and Methods

### Phylogenomic dataset for introgression analyses

We used phylogenomic data from a recent study (Vargas *et al*., 2020) that produced a highly resolved phylogeny for American *Costus*, with the primary objective of understanding the drivers of speciation (i.e. vertical inheritance) within the radiation. By contrast, our study focuses on interspecific gene flow (i.e. horizontal inheritance) to investigate the role of reticulate evolution in the diversification of American *Costus*. Briefly, Vargas *et al*. (2020) employed an exon‐targeted sequencing approach to generate 756 single‐copy orthologous genes across 113 samples representing 57 distinct *Costus* taxa (54 American taxa and three African outgroup species). The average length per gene, including partial introns, was 1951 bp, and each sample, on average, was represented in 728 of 756 total sequence alignments. Vargas *et al*. (2020) inferred the *Costus* species tree with this dataset using both coalescent and concatenation‐based approaches, which yielded highly supported and topologically similar phylogenies. Because individual gene trees exhibited low‐phylogenetic signal, we use the concatenation topology in this study. The concatenation topology was inferred with IQ‐Tree (Nguyen *et al*., 2015) with an independent GTR + G model of sequence evolution for each gene partition and 1000 ultrafast bootstraps. This topology was used as a starting tree for phylogenetic network optimization with SNaQ and PhyloNet, served as the species phylogeny for generating species configurations for the gene‐tree‐ and site‐pattern‐based tests, and was used for visualizing results of introgression analyses. American *Costus* is undergoing taxonomic revision and thus some species names in this study are likely to change. We provide details of taxonomy, collection locations, and voucher information in Supporting Information Table S1.

### Inferring introgression across the American *Costus* radiation

We used four phylogenetic approaches to detect introgression. First, we conducted rooted triplet (RT) tests (Larson *et al*., 2021) on multiple three taxa subsets from the phylogeny. This test compares the proportions of loci that support each of the three possible topologies for a given set of three taxa and an outgroup. Under the multispecies coalescent, the most common topology is expected to reflect the true species relationship (the major relationship), and the two less common topologies (minor relationships) are expected to result from incomplete lineage sorting (ILS). Assuming a null hypothesis of ILS only and no admixture, the two minor relationships should be equally frequent within the distribution of gene trees, and statistically significant deviations from equality may be considered evidence for gene flow. For each rooted triplet, each gene alignment was pruned to include only the three ingroup taxa of interest and the outgroup taxon, *C. lucanusianus*, which is a member of the distant African clade of *Costus*. The gene trees were estimated with IQ‐Tree (Nguyen *et al*., 2015). *P*‐values for the tests were calculated by comparing the observed distributions of minor topologies to a binomial distribution with the probability of either minor relationship being 0.5. To generate the set of triplets to test, we sampled each pair of sister species from the tree and paired them with each of the remaining samples, resulting in 1247 input tests. To account for multiple tests using the same taxa, we adjusted the *P*‐values with the Holm–Bonferroni method (Holm, 1979) using the *P.adjust* function in R (R Core Team, 2024) with a significance threshold of 0.001. This method does not attempt to mitigate the effects of gene tree estimation error (GTEE), but we nonetheless expect our false positive rate to be low given the large number of gene trees that informed each test (average number of genes per test = 687) and conservative significance threshold (Holm–Bonferroni FWER < 0.001). For each significant test, we estimated the proportion of the genome that was inherited during hybridization (*γ*) with the following equation from Suvorov *et al*. (2022): *γ* = (minor_2_ – minor_1_)/(major + minor_1_ + minor_2_). In this equation, major, minor_1_, and minor_2_ represent the counts of genes reflecting the major relationship and the two minor relationships, where minor_2_ > minor_1_. In cases where sets of tests shared a common ancestor, potentially indicating hybridization along internal branches, we estimated the introgression proportions using a method analogous to the f‐branch metric (eqn 6 in Malinsky *et al*., 2021), as described in more detail below. The RT tests were conducted with previously published Python scripts (Larson *et al*., 2021) and their software dependencies, including iq‐tree and phyx (Nguyen *et al*., 2015; Brown *et al*., 2017). For all tests, the gene trees were rooted with *C. lucanusianus*.

In addition to RT tests, we calculated *D*‐statistics for all four‐taxon subsets using dsuite (Malinsky *et al*., 2021). The *D*‐statistic, commonly known as the ABBA‐BABA test (Green *et al*., 2010; Patterson *et al*., 2012), compares the frequencies of ABBA and BABA site patterns across three species and an outgroup, where ‘A’ represents the ancestral allele and ‘B’ the derived allele. The related *f*_4_‐ratio (Patterson *et al*., 2012) estimates the proportion of the genome inherited via introgression using these ABBA and BABA frequencies. To perform these calculations, we first concatenated 756 single‐gene multiple sequence alignments into a single FASTA file using catfasta2phyml (https://github.com/nylander/catfasta2phyml) with the *–concatenate* argument. We then analyzed 24 804 unique combinations of three species using a custom Python script (run Dsuite.py). For each combination, SNPs were extracted from the concatenated FASTA file using SNP‐sites (Page *et al*., 2016), filtered for biallelic sites with vcftools v.1.13 (Danecek *et al*., 2011), and thinned to one every 500 base pairs, resulting in an average of 2605 SNPs (SD = 40) per combination. We used the Dsuite *Dtrios* command to calculate *D*‐statistics, with *C. lucanusianus* as the outgroup to infer the ancestral state. Significance was assessed via block‐jackknifing with 40 blocks, each containing an average of 64 variants. Hybridization along internal branches can produce correlated, elevated *D*‐statistics and *f*_4_‐ratio scores, making interpretation challenging. To address this, Malinsky *et al*. (2018) developed the f‐branch metric, which detects excess allele sharing between a tip (P3) and a particular internal branch (b). This is done by taking the median of the minimum *f*_4_‐ratios between P3 and the descendants of branch (b), calculated across all descendants of the sister branch to (b). Because calculation of the f‐branch metric relies on a particular tree hypothesis, we used the Dsuite *Dtrios* output file with the ‘tree.txt’ suffix, which contains the *D*‐statistics for species trios that were arranged to be consistent with the maximum likelihood *Costus* phylogeny (Vargas *et al*., 2020). The *f*_4_‐ratio and f‐branch metrics typically rely on allele frequencies within each species. However, since most species in our dataset had only 1–2 samples, we used introgression proportions calculated from rooted triplet gene tree topologies instead of *f*_4_‐ratios. For each significant *D*‐statistic (Holm–Bonferroni *P*‐value < 0.001), we conducted a corresponding rooted triplet (RT) test and replaced the *f*_4_‐ratio with an introgression proportion (*γ*) calculated as:

$$\gamma =\frac{\left({\text{minor}}_{2}-{\text{minor}}_{1}\;\right)}{\left(\text{major}+{\text{minor}}_{1}+{\text{minor}}_{2}\;\right)}$$

where major, minor_1_, and minor_2_ represent the gene counts for the major and two minor topologies, respectively, with minor_2_ > minor_1_. The f‐branch metric was computed using these introgression proportions with the Dsuite *Fbranch* command and visualized using dtools.py from the dsuite package.

For pairs of taxa exhibiting a significant signal of introgression, we used divergence‐based introgression polarization (DIP) analyses (Forsythe *et al*., 2020a,b) to test for asymmetric introgression and to polarize the direction of introgression. The basic implementation of DIP (referred to as 1 × DIP) is based on the expectation that sequence divergence between the recipient taxon and its sister taxon increases due to introgression from a donor taxon. To distinguish between asymmetric and symmetric introgression, DIP calculates the pairwise sequence divergence among two sister taxa (P1 and P2) and a third taxon (P3) that hybridizes with P2. Two additional analyses, double‐DIP (2 × DIP) and triple‐DIP (3 × DIP), include additional steps that allow detection of asymmetry in cases of bidirectional introgression and correction of gene tree classification biases caused by ILS. We applied the 1×, 2×, and 3 × DIP analyses to the set of taxon pairs for which introgression was detected based on significant RT tests.

In addition, we used model‐based phylogenetic network approaches to identify putative introgression events in *Costus*. SNaQ (Solís‐Lemus & Ané, 2016; Solís‐Lemus *et al*., 2017) implements maximum pseudolikelihood optimization to estimate network topology, branch lengths, and inheritance probabilities (*γ*) based on a table of quartet concordance factors and an initial starting tree as input. Because optimization can be prohibitively slow for large networks, we ran SNaQ on four subsets of the dataset containing between 13 and 21 species. Before calculating quartet concordance factors, we collapsed gene tree branches with < 90% ultrafast‐bootstrap support. For each taxon subset, we estimated four networks where the maximum number of hybridizations (*h*) ranged from zero to three. Each network was estimated with twenty independent runs, and a slope heuristic was used to identify the best *h* value (Solís‐Lemus & Ané, 2016). The maximum pseudolikelihood networks were visualized with PhyloPlots included with the phylonetworks julia package (Solís‐Lemus *et al*., 2017).

In addition to SNaQ, phylogenetic networks were inferred with the maximum pseudolikelihood inference method included in PhyloNet (Than *et al*., 2008; Yu & Nakhleh, 2015). To ease computational intensity, we split the samples into four‐taxon subsets and used a tree‐based augmentation approach whereby a previously inferred maximum likelihood *Costus* phylogeny (Vargas *et al*., 2020) was fixed as the backbone tree and augmented into a network using the *‐fs* option with the *InferNetwork MPL* method. To generate gene trees as input for PhyloNet, we used RAxML‐NG (Kozlov *et al*., 2019) to infer maximum likelihood gene trees and bootstrap supports under the GTR + G model of rate heterogeneity and 900 nonparametric bootstrap replicates. Gene tree branch lengths were removed in accordance with PhyloNet best practices (Cao *et al*., 2019). To assess the influence of using different bootstrap thresholds when contracting poorly supported tree edges, we independently analyzed three bootstrap support thresholds (70, 80, and 90%) per taxon subset and compared the results of each. Networks were inferred with 500 search runs (−×500) for 0, 1, 2, and 3 maximum number of reticulations. A slope heuristic approach (Solís‐Lemus & Ané, 2016) was used to identify the best maximum number of reticulations for each bootstrap threshold and taxon subset. All networks were visualized with PhyloPlots included with the phylonetworks julia package (Solís‐Lemus *et al*., 2017).

## Results

**Fig. 1:**
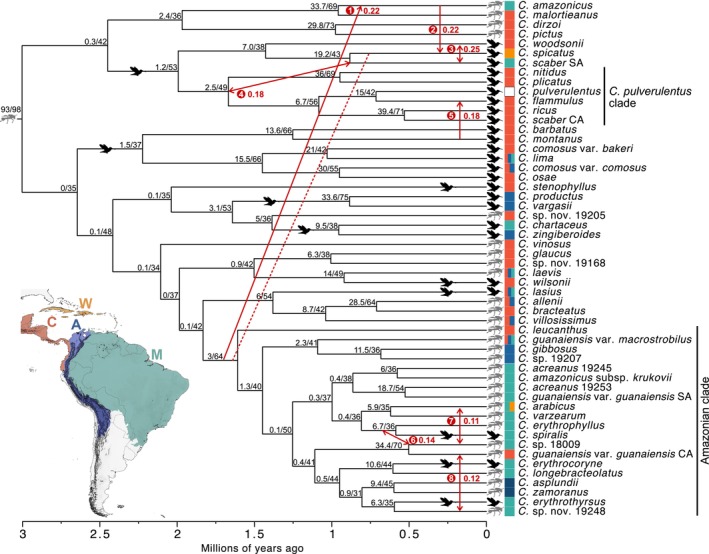
A visual summary of hybridization events in *Costus* inferred with RT tests. Events are indicated by solid red arrows, and red circles provide the numeric label for each event as it is referenced in the text and Table 1. The event indicated with a dotted line was detected but may have resulted from events 1 and 2 rather than from hybridization. When calculation was possible, arrows were annotated with *γ*, an estimate of the proportion of the genome that was inherited via introgression. When asymmetric introgression was detected, a single arrowhead indicates the predominant direction. Bee/hummingbird symbols and colored boxes along the tips of the phylogeny indicate the pollination syndromes and geographic ranges for each taxon. Four geographic areas are defined (A, Andes; C, Central America; M, Amazon; W, West Indies). Boxes with two or more colors represent geographic ranges that span two or more areas. A white box indicates a geographic range that spans all four areas. Hummingbird symbols that occur along branches indicate putative evolutionary shifts from bee‐ to hummingbird pollination. For some polyphyletic taxa, the accessions are distinguished geographically (CA, Central American; SA, South American). Nodes are labelled with the gene and site concordance factors, separated by a forward slash (/), indicating the fraction of decisive gene trees and alignment sites supporting each branch, respectively.

**Fig. 2:**
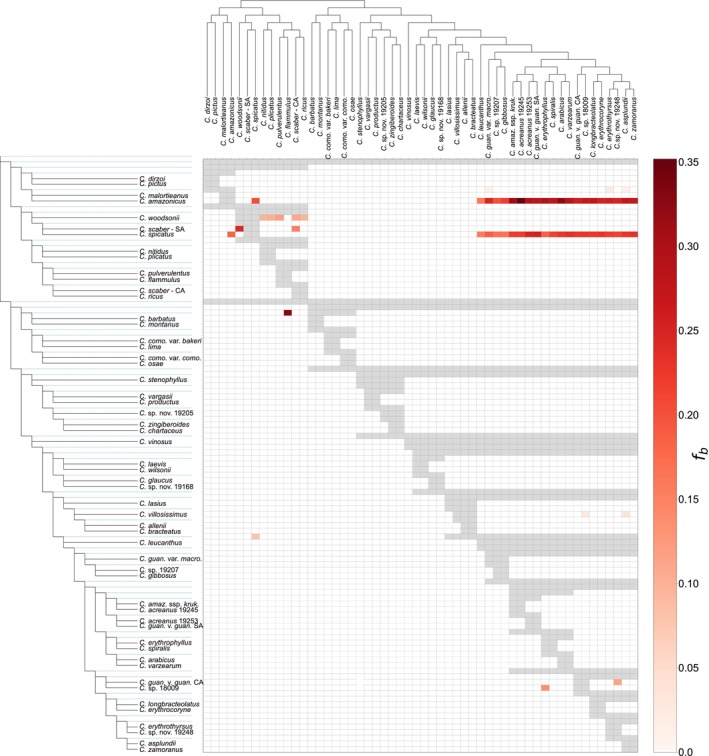
A visual summary of f‐branch metrics reflecting evidence of interspecific gene flow in *Costus*. The f‐branch metric (Malinsky *et al*., 2018, 2021) identifies excess allele sharing between species (*x*‐axis) and the descendants of a branch (*y*‐axis) that may have resulted from gene flow. The tree along the *y*‐axis is ‘expanded’, so that each branch, including internal branches (depicted with blue dotted lines), is shown. Excess allele sharing between species and branches is denoted with red shading, with darker shades of red indicating higher levels of interspecific gene flow. Grey shading indicates combinations of branches and species where *f*‐branch metrics could not be calculated due to the lack of a suitable sister branch.

We detected a signal of hybridization in a subset of species pairs using the RT test method. Evidence of introgression was detected in 55 species pairs, with a family‐wise error rate (FMER) of < 0.001 after applying the Holm–Bonferroni correction. After collapsing correlated tests sharing a common ancestor, indicating an ancient introgression event, we inferred nine putative hybridization events. However, based on patterns of asymmetric introgression inferred with SNaQ, PhyloNet, and DIP, it is likely that the signal for one of these events (hybridization between *C. spicatus* and the ancestor of the Amazonian clade; Fig. 1, dashed line) was produced indirectly through introgression from the ancestor of the Amazonian clade into *C. amazonicus* (event 1; Fig. 1) and introgression from *C. amazonicus* into *C. spicatus* (event 2; Fig. 1). As a result, we have excluded this event from Table 1. We inferred three events that involved ancestral lineages (events 1, 4, and 6; Fig. 1; Table 1) and five among terminal taxa (events 2, 3, 5, 7, and 8; Fig. 1; Table 1). All but one event was between species or lineages with matching pollination syndromes (e.g. both hummingbird‐ or bee‐pollinated; Table 1). In only half of the events did the species or lineages co‐occur in the same broad geographic zone (West Indes, Central America, Andes, Amazon; Table 1; Fig. 1). For the three cases of ancient hybridization, previously published ancestral reconstructions of pollination syndrome and geographic range (Vargas *et al*., 2020) were used (Table 1). Finally, four of the eight events inferred with RT tests were also inferred with the f‐branch metric, two events were partially consistent with f‐branch metrics, and two events were not concordant with f‐branch metrics (Table 1; Figs 1, 2).

**Table 1:** Summary of hybridization events in *Costus* detected with RT tests.

Event	Lineages involved	Detected with f‐branch?	Age range (Ma)	Pollination syndromes	Geographic areas
1	Ancestor of Amazonian clade → *C. amazonicus*	Yes	2.01–0.83	Bee–bee	Amazon–Amazon
2	*C. amazonicus* → *C. spicatus*	Yes	1.75–present	Bee–bird	Amazon–West Indes
3	*C. woodsonii* ↔ *C. scaber* (SA)	Yes	1.75–present	Bird–bird	CA–Amazon
4	*C. scaber* (SA) ↔ ancestor of *C. pulverulentus* clade	No	1.75–0.7	Bird–bird	Amazon–CA
5	*C. montanus* → *C. flammulus*	Partial	1.42–present	Bird–bird	CA–CA
6	Ancestor of *C. erythrophyllus* and *C. spiralis* ↔ *Costus* sp. 18009	Partial	1.01–0.16	Bee–bee	Amazon–Amazon
7	*C. arabicus* ↔ *Costus* sp. 18009	No	1.01–present	Bee–bee	Amazon–Amazon
8	*C. guanaiensis* var. *guanaiensis* CA ↔ *Costus* sp. nov. 19248	Yes	1.01–present	Bee–bee	CA–Amazon

This table provides additional information about each hybridization event, including whether the event was also detected with f‐branch metrics summarizing significant *D*‐statistics (Fig. 2; Table S3). Event numbers correspond to the text and the numbered arrows in Fig. 1. Arrows depict gene flow between lineages, with single arrowheads indicating the primary direction when evidence for asymmetric introgression was found. The projected age ranges for when hybridization occurred are based on node age estimates from Vargas *et al*. (2020). The pollination syndromes and geographic ranges associated with the hybridizing taxa are also provided. When hybridization involved an ancestral lineage, ancestral reconstructions of pollination syndrome and geographic ranges were used from Vargas *et al*. (2020). CA, Central America; SA, South America.

In addition to RT tests, we used dsuite to calculate *D*‐statistics for all possible trios of taxa. After adjusting for multiple tests with the Holm–Bonferroni correction, 141 species pairs were significant compared to 55 species pairs recovered with RT tests. We analyzed the results of the *D*‐statistic tests with the f‐branch metric in dsuite to examine patterns of excess allele sharing between internal branches that may indicate ancient introgression (Fig. 2). The *D*‐statistic and f‐branch analyses were slightly less conservative than the RT test analyses and detected more potential introgression events. However, many of the events detected with *D*‐statistics but not RT tests possessed low f‐branch values (f‐branch < 0.04), suggesting low levels of gene flow (Fig. 2; Table S3). Many significant *D*‐statistic tests exhibited a pattern whereby the ABBA site pattern frequency was greater than the BBAA site pattern frequency (i.e. the site pattern caused by gene flow was more common than the site pattern caused by vertical inheritance from a common ancestor). We discuss the drivers and implications of this pattern in the Notes S1.

The basic (1 × DIP) and extended implementations (2 × DIP and 3 × DIP) of DIP were conducted to polarize the direction of introgression events detected with the RT test analyses. Due to the likelihood of high rates of ILS caused by rapid and recent diversification in *Costus*, we report only the results of the 3 × DIP analyses here, which correct for biases caused by gene tree ILS. Detailed reporting of the 3 × DIP results and a table with the results of the 1 × DIP and 2 × DIP analyses can be found in the Table S2; Notes S2. When statistically significant evidence for asymmetric introgression was detected with DIP, the predominant directions were denoted with unidirectional arrows in Fig. 1. Otherwise, a lack of evidence for asymmetric introgression is depicted with bidirectional arrows. The 3 × DIP analyses provided evidence for asymmetric introgression for three of the events inferred with RT tests: (1) from the ancestor of the Amazonian clade into *C. amazonicus*, (2) from *C. amazonicus* into *C. spicatus*, and (3) from *C. montanus* into *C. flammulus*.

**Fig. 3:**
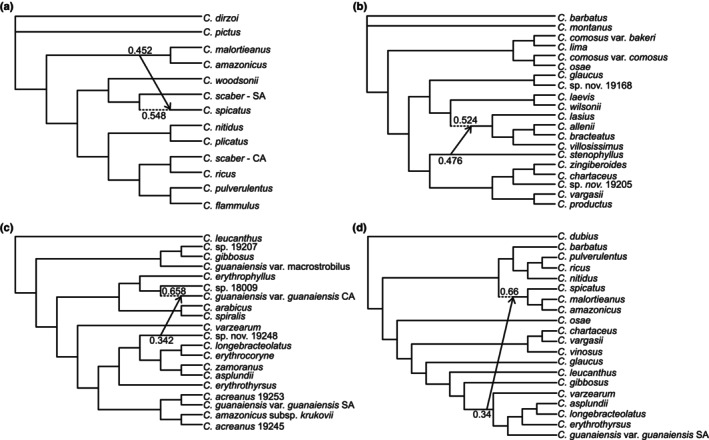
Best supported phylogenetic networks for *Costus* inferred with SNaQ for three separate clades (a–c) and a subset of taxa that spans the entire phylogeny (d). Minor hybrid edges are represented by arrows and annotated with inheritance probabilities (*γ*) at their origin. Major hybrid edges are represented by dashed lines and are annotated with 1–*γ*, which is an estimate of the proportion of the genome inherited vertically from an ancestor. Only the networks with the number of hybridization events (*h*) that provided the best fit for each subset are shown here, but all networks for *h* = 0–3 are provided in Supporting Information Figs S2–S5.

Due to computational limitations, we conducted separate SNaQ analyses on four subsets of taxa. For each of the four subsets, the network scores exhibited the sharpest decline from *h* = 0 to *h* = 1 (Fig. S1), suggesting one hybridization provided the best fit for all subsets based on a slope heuristic. The first subset included a clade that diverged early and diversified largely in Central America (Fig. S2). The major trees in the networks with *h* = 1–3 were identical to the concatenation‐based species tree in Vargas *et al*. (2020), with South American *C. scaber*, *C. spicatus*, and *C. woodsonii* as monophyletic and South American *C. scaber* and *C. spicatus* as sister (Fig. S2). With *h* ≥ 1, a hybrid edge from the ancestor of *C. amazonicus* and *C. malortieanus* into *C. spicatus* was consistently inferred, and the proportion of genes inherited via hybridization (*γ*) was estimated as 0.45 (Figs 3a, S2). The other three analyses (RT tests, *D*‐statistic/f‐branch, and PhyloNet) detected similar events between *C. amazonicus* and *C. spicatus* (Figs 1, 2). The network with *h* = 0 was nearly identical to the species tree but with south American *C. scaber*, *C. spicatus*, and *C. woodsonii* as paraphyletic and *C. spicatus* closer to the *C. amazonicus–C. malortieanus* clade (Fig. S2). This discordance can be explained by the *h* = 0 network not accounting for hybridization involving *C. spicatus*, which was inferred in all networks where *h* ≥ 1 (Figs 3a, S2).

The second subset of taxa analyzed with SNaQ included a grade that connects the first and third subsets (Fig. S3). The network with *h* = 0 was identical to the species tree in Vargas *et al*. (2020), and the major tree in the network with *h* = 1 was nearly identical to the species tree but for *C. glaucus*, *Costus* sp. nov. 19168, *C. laevis*, and *C. wilsonii* as paraphyletic (Fig. 3b). With *h* ≥ 1, a hybrid edge from *C. stenophyllus* into the ancestor of *C. lasius*, *C. villosissimus*, *C. allenii*, and *C. bracteatus* was consistently observed (*γ* = 0.48) (Fig. 3b). This event was not recovered by any of the other analyses.

The third subset of taxa analyzed by SNaQ was the Amazonian clade (see label in Figs 1, S4). There was substantial topological variation among the species tree and the networks inferred for all values of *h* (though variation among networks was relatively less pronounced) (Fig. S4). Lack of congruence among the species tree and the networks may be explained by high levels of gene tree discordance (see Fig. 1 in this manuscript and Fig. S3 In Vargas *et al*., 2020) in this particularly young clade (< 2 ma; Vargas *et al*., 2020). With *h* = 1, a hybrid edge from *Costus* sp. nov. 19248 into central American *C. guanaiensis* var. *guanaiensis* was inferred (*γ* = 0.34) (Figs 3c, S4), consistent with the results of the RT tests and *D*‐statistics/f‐branch (Figs 1, 2).

The final subset of taxa analyzed by SNaQ spanned the major splits in American *Costus* (Fig. S5). The network with *h* = 0 was similar to the species tree, except for the placements of *C. barbatus* and *C. spicatus* (Fig. S5). With *h* = 1, a hybrid edge (*γ* = 0.34) was inferred from the ancestor of an Amazonian subclade (*C. varzearum*, *C. asplundii*, *C. longebracteolatus*, *C. erythrothyrsus*, and *C. guanaiensis* var. *guanaiensis*) to the ancestor of *C. spicatus*, *C. amazonicus*, and *C. malortieanus* (Fig. 3d). The incorrect placement of *C. spicatus* with *C. amazonicus* and *C. malortieanus* (Fig. 3d) can be explained by strong introgression from *C. amazonicus* and/or *C. malortieanus* into *C. spicatus* (*γ* = 0.34, Fig. 3a). With *h* ≥ 2, the hybrid edge (*γ* = 0.4–0.41) (Fig. S5) was inferred from the Amazonian subclade into *C. amazonicus* only, which supports the scenario in which only *C. amazonicus*, and not *C. spicatus*, hybridized with an Amazonian ancestor.

Phylogenetic networks were additionally inferred with the pseudolikelihood method of PhyloNet. Contracting poorly supported gene tree edges according to different bootstrap support thresholds (70, 80, and 90%) influenced the resulting networks, including the best number of reticulations and the inferred topologies. For all taxon subsets, network inference using the gene trees contracted with the most conservative bootstrap threshold (90%) did not yield evidence for introgression, suggesting these datasets lacked sufficient information to resolve reticulation edges. By contrast, datasets produced using the more liberal bootstrap thresholds (70 and 80%) provided support for the addition of reticulation edges in three of the four‐taxon subsets using a slope heuristic approach to assess the best‐fitting model (Fig. S6). In clade 1, the addition of a single reticulation edge was supported using a bootstrap threshold of 70% (Fig. S6). This edge, from the ancestor of *C. malortieanus* and *C. amazonicus* into the ancestor of *C. spicatus* and South American *C. scaber*, is similar but not identical to reticulation events inferred by RT tests, *D*‐statistic/fbranch analyses, and SNaQ (Fig. S7A). In grade 2, no reticulation edges were supported based on a slope heuristic (Figs S6, S8). In clade 3, the addition of two reticulation edges was supported using a bootstrap threshold of 80% (Fig. S6). However, both edges had low‐inheritance probabilities and were not corroborated by other methods (Fig. S9B). In subset 4, two and one reticulation edges were inferred using bootstrap thresholds of 70% and 80%, respectively (Fig. S6). One of these edges, from the ancestor of *C. ricus* and *C. pulverulentus* into *C. barbatus*, may be consistent with hybridization between *C. barbatus* and/or *C. montanus* and *C. flammulus*, since neither *C. flammulus* nor *C. montanus* was included in this analysis (Fig. S10A). However, the 3 × DIP analyses suggest asymmetric introgression from *C. montanus* into *C. flammulus*, which is opposite to the direction inferred by PhyloNet. Further details on PhyloNet results and the corresponding phylogenetic networks are provided in the Figs S6–S10 and Notes S3.

## Discussion

Our study provides the first clade‐wide view of introgression throughout the evolutionary history of *Costus*, which has experienced a rapid and recent radiation in the Central and South American tropics. Recent studies have provided robust phylogenies for *Costus* (Vargas *et al*., 2020; Valderrama *et al*., 2022), but we have thus far lacked an understanding of the frequency and extent of introgression within this diverse and interfertile clade. By providing a consensus of putative instances of introgression, our results advance our understanding of hybridization and the factors that underlie observed patterns in evolutionary radiations.

### Patterns of introgression across the *Costus* radiation

Our independent approaches largely converged on similar sets of taxon pairs exhibiting signatures of introgression. Due to differences in sensitivity, the analyses varied in the frequency of introgression inferred, which we discuss below. We detected introgression between eight pairs of taxa using a gene‐tree‐based method (RT test) after collapsing significant events indicating ancestral introgression. These results suggest that the frequency of introgression in *Costus* is comparable to, and occasionally lower than, what has been observed in other tropical plant groups studied to date. For example, Larson *et al*. (2021) detected admixture in two species pairs within a clade of 12 Amazonian rainforest tree species (*Eschweilera*; Parvifolia clade). Morales‐Briones *et al*. (2018b) found evidence of at least 24 hybrid species in a study of 46 *Lachemilla* species. Vargas *et al*. (2017) reported at least 12 reticulation events among 36 species of *Diplostephium* and related taxa, while Gardner *et al*. (2023) uncovered at least 5 reticulation events in a study of 42 *Ficus* species.

Hybridization may be common in evolutionary radiations, as divergence often occurs in sympatry or parapatry and outpaces the development of reproductive isolation (Seehausen, 2004; Pontarp *et al*., 2024). Consistent with this expectation, widespread hybridization and introgression have been found in many rapid radiations, including *Heliconius* butterflies (Edelman *et al*., 2019), Malawi cichlids (Malinsky *et al*., 2018), and Darwin's finches (Lamichhaney *et al*., 2015). Like other radiations, the evolutionary history of *Costus* has been shaped by introgression. However, our analyses revealed less introgression than one may expect given the rapid rate of speciation and high degree of interfertility across the genus. Additionally, rates of hybridization appear to vary across the genus. For example, introgression was notably rare in the Amazonian clade and the grade of species that subtends it. Although the Amazonian clade has undergone a particularly recent and rapid burst of speciation, we recovered evidence for only three reticulation events, all of which were associated with relatively small introgression proportions (Figs 1, 2).

Our analyses recovered evidence for introgression during both the early and later stages of *Costus* diversification. Almost half of the hybridization events involved at least one ancestral lineage (i.e. a common ancestor of a set of present‐day species). We resolved a particularly ancient event between the *C. amazonicus* lineage and the ancestor of the Amazonian clade. Based on a recent time calibration of the *Costus* phylogeny (Vargas *et al*., 2020), the ancestor of the Amazonian clade arose *c*. 1.8 Mya (CI = 0.9–2.8) and the *C. amazonicus* lineage arose *c*. 1 Mya (CI = 0.2–2), suggesting that hybridization between *C. amazonicus* and the Amazonian clade ancestor likely occurred at least 1 million years ago (Ma), representing a significantly deep reticulation in the *c*. 3 Ma old American *Costus* clade (Vargas *et al*., 2020). Evidence for deep histories of reticulation have been uncovered in other plant clades, including *Lachemilla* (Rosaceae) (Morales‐Briones *et al*., 2018a), *Brownea* (Fabaceae) (Schley *et al*., 2020), and *Ficus* (Moraceae) (Gardner *et al*., 2023). Currently, phylogenetic network model approaches provide the most straightforward method for inferring introgression involving ancestral lineages because they allow for the estimation of hybrid edges along internal branches. Alternatively, individual three and four‐taxon tests can not directly test for introgression involving ancestral lineages but sets of significant tests in the descendants of the same common ancestor can provide robust evidence (Malinsky *et al*., 2021). Utilizing both model‐based and test‐based methods, we recovered largely parallel evidence for ancient introgression, suggesting both approaches are valid for resolving deep reticulation.

The degree of asymmetry in gene flow and its dominant direction can inform our understanding of reproductive isolation in pairs of lineages in which introgression occurs. We found evidence for asymmetric introgression in three of the eight events inferred with the RT tests, consistent with reproductive isolating barriers preventing backcrossing with one of the parental taxa. Strong asymmetric introgression from the ancestor of the Amazonian clade into *C. amazonicus* and from *C. amazonicus* into *C. spicatus* explains why a large proportion of gene trees support a sister relationship between *C. spicatus* and members of the Amazonian clade. In this case, *C. amazonicus* likely acted as a bridge by allowing genetic transfer from the ancestor of the Amazonian clade to *C. spicatus* without direct hybridization. Similar processes have likely occurred in other parts of the phylogeny, as evidenced by pairwise f‐branch metrics (Fig. 2), showing excess allele sharing between *C. woodsonii* and five of the six members of the *C. pulverulentus* clade. Rather than resulting directly from introgression between *C. woodsonii* and the members or ancestor of the *C. pulverulentus* clade, it is possible that South American *C. scaber* acted as a bridge, facilitating gene flow between *C. woodsonii* and the *C. pulverulentus* clade. It is unclear how often indirect signals of introgression arise in the literature due to promiscuous taxa hybridizing with two or more taxa, but our results urge caution in interpretation and underscore the importance of using clade‐wide studies if the goal is to identify putative cases of introgression or estimate the frequency of hybridization.

### Possible causes of limited introgression in American *Costus*

Despite widespread interfertility throughout American *Costus*, we inferred limited introgression and conclude that incomplete lineage sorting, and not reticulation, is the primary driver of gene tree discordance across the phylogeny. For example, gene tree discordance was highest in the Amazonian clade (Fig. 1 in this manuscript and Fig. S3 in Vargas *et al*., 2020), which has experienced a recent burst of diversification in the Amazon basin, but our results suggest that rates of introgression are similar or lower in the Amazonian clade than in other clades. The low incidence of introgression in American *Costus* may be explained by strong barriers to hybridization where species co‐occur, consistent with strong ecological divergence and/or reinforcement. During evolutionary radiations, where rapid speciation requires swift reproductive isolation, extrinsic barriers such as ecological divergence and geographic isolation are expected to play a larger role than the slower accumulation of intrinsic genetic incompatibilities (Gillespie *et al*., 2020). In *Costus*, floral isolation is a strong reproductive barrier between sympatric species (Kay & Schemske, 2003), and our results suggest that floral isolation has also influenced patterns of introgression across the evolutionary history of *Costus*. Seven of the eight instances of introgression inferred with RT tests were between taxa or ancient lineages that shared pollination syndromes: four between bee‐pollinated species, three between hummingbird‐pollinated species, and one between species with contrasting pollination syndromes (Table 1). A recent phylogenomic study of the magic flowers (*Achimenes*) also detected hybridization in only two pairs of species, both of which exhibited matching pollination syndromes (Roberts & Roalson, 2018), and many studies have documented an important role of floral specialization in reproductive isolation in a variety of plant groups, including orchids (Moccia *et al*., 2007; Xu *et al*., 2011; Whitehead & Peakall, 2014), figs (Wang *et al*., 2016; Satler *et al*., 2023), and monkeyflowers (Sobel & Streisfeld, 2015).

Reinforcement of reproductive isolation may have also reduced introgression in *Costus* (Kay & Schemske, 2008). Theoretical work suggests that reinforcement is most likely under conditions of low gene flow between diverging taxa since the homogenizing effects of gene flow and recombination can prevent the evolution of prezygotic isolating mechanisms (Butlin, 1987). A well‐studied example of reinforcement in *Costus* is between Central American *C. scaber* and *C. pulverulentus*, where locally sympatric populations exhibit strong prezygotic pollen–pistil incompatibilities but allopatric populations are fully interfertile (Kay & Schemske, 2008). Previous population genetic data revealed that though they co‐occur and share hummingbird pollinators, hybridization is rare, and levels of introgression are low (interspecific ancestry comprised > 5% of the genome in only 3.8% of individuals) (Surget‐Groba & Kay, 2013). In the current study utilizing phylogenomic data, we did not detect introgression between Central American *C. scaber* and *C. pulverulentus*, suggesting that either the signal of introgression was absent or too low to be detected with our methods (the sensitivity of our analyses is discussed further below). A lack of evidence for gene flow between Central American *C. scaber* and *C. pulverulentus* in our analyses suggests that reinforcement in contact zones, initially driven by low levels of interspecific gene flow in sympatric populations, has effectively limited introgression at the species level.

### Potential sources of bias and discordance

Despite a large degree of consistency, the methods differed in the number of events detected. The *D*‐statistic analyses exhibited the highest sensitivity, detecting a signal of introgression in 46 of 55 (83.6%) species pairs detected by RT tests and an additional 95 species pairs. A similar disparity was also observed in a recent study (Suvorov *et al*., 2022), where a site‐pattern‐based analysis (HyDe) identified 142 of 152 (93.4%) species pairs detected by both gene‐tree‐based approaches (DCT and BLT) and 898 additional species pairs. One potential explanation for this disparity is that gene‐tree‐based tests have lower false positive rates than site‐pattern‐based tests, though this hypothesis has not been directly tested to our knowledge. The phylogenetic network approaches, SNaQ and PhyloNet, were less sensitive than the four‐taxon tests, though many of the reticulation events inferred with the four‐taxon tests were likewise inferred with phylogenetic networks.

For the youngest taxon subsets (clade 3 and subset 4), the phylogenetic networks inferred with SNaQ and PhyloNet diverged from the previously inferred species tree (Vargas *et al*., 2020) and the introgression scenarios proposed by the test‐based methods. This discordance between the test‐based and phylogenetic network methods may stem from a lack of identifiability of certain introgression scenarios or of the underlying species tree topology, especially in the presence of high ILS, and suggests caution when analyzing complex phylogenetic histories with model‐based approaches alone. Additionally, the phylogenetic networks diverged from the test‐based methods in their inferred admixture proportions, which were overall higher in the networks. As a notable example, the admixture proportions associated with introgression from *C. amazonicus* into *C. spicatus* were estimated as 0.22 with the RT test analysis but were 0.45 and 0.42 with the SNaQ and PhyloNet phylogenetic network inference approaches, respectively. As phylogenetic network methods are still relatively new, the number of studies that provide a comparison of phylogenetic networks and test‐based analyses is limited (but see Kleinkopf *et al*., 2019), precluding a deeper investigation into the basis for this discrepancy. Nonetheless, this pattern suggests that the source of the discrepancy may lie in the intrinsic differences between test‐based and model‐based methods.

One potential limitation in this study is the lack of power to detect introgression when small proportions of the genome were horizontally inherited, possibly leading us to underestimate the frequency of hybridization. For the majority of the events, estimates of the extent of introgression and the mean proportion of the genome affected were large (RT tests: 18% [SD = 5%]; SNaQ: 32% [SD = 11%]), though within the range of other plants (Morales‐Briones *et al*., 2018a; Schley *et al*., 2020; Rose *et al*., 2021) and some animals (MacGuigan & Near, 2019; Suvorov *et al*., 2022). Future studies using whole genome sequences or other loci may uncover additional events, but we expect these events to have a small genomic footprint. Subsequent population genomic analyses may also uncover additional events between sister taxa that our analyses were unable to detect. Additionally, incomplete sampling of extant and extinct lineages (i.e. ‘ghost’ lineages) is unavoidable and may have led us to wrongly identify the donors and recipients of introgression (Tricou *et al*., 2022). Although the sampling effort for this study is high based on our contemporary understanding of *Costus* diversity, it is incomplete. Whereas future studies will undoubtedly incorporate additional taxa and refine our understanding of introgression in American *Costus*, a more intractable issue is the influence of extinct lineages, which is unlikely to improve as our taxonomic knowledge increases.

### Conclusions

This study contributes to the growing body of research on the prevalence of hybridization and reticulate evolution. Our results demonstrate the utility of targeted sequencing data for detecting introgression within young species radiations with substantial gene tree discordance. Our phylogenomic analyses provided multiple sources of evidence that introgression has occurred at least eight times during the evolutionary history of American *Costus*. Moreover, our investigations revealed evidence of ancient reticulation events involving the ancestors of modern‐day species, in addition to more recent events. Consistent with our expectations, hybridization was almost exclusively detected between taxa that share pollination syndromes, either orchid bee or hummingbird. These findings suggest that prezygotic reproductive isolating barriers have significantly influenced patterns of hybridization in *Costus*, further highlighting the critical role of such barriers in the diversification of this tropical understory plant radiation.

## Competing interests

None declared.

## Author contributions

OMV, KMK and KAU designed the project and collected the data. KAU led the data analysis with input from OMV and KMK. KAU wrote the manuscript with input from OMV and KMK.

## Supporting information


**Fig. S1** Network scores for SNaQ analyses.
**Fig. S2** Phylogenetic networks for clade 1 inferred with SNaQ.
**Fig. S3** Phylogenetic networks for grade 2 inferred with SNaQ.
**Fig. S4** Phylogenetic networks for clade 3 inferred with SNaQ.
**Fig. S5** Phylogenetic networks for subset 4 inferred with SNaQ.
**Fig. S6** Network scores for PhyloNet analyses.
**Fig. S7** Phylogenetic networks for clade 1 inferred with PhyloNet.
**Fig. S8** Phylogenetic networks for grade 2 inferred with PhyloNet.
**Fig. S9** Phylogenetic networks for clade 3 inferred with PhyloNet.
**Fig. S10** Phylogenetic networks for subset 4 inferred with PhyloNet.
**Notes S1** Site pattern frequencies in significant vs nonsignificant *D‐*statistic tests.
**Notes S2** DIP analyses to detect and polarize asymmetric introgression.
**Notes S3** Pseudolikelihood phylogenetic network inference with PhyloNet.
**Table S1** List of samples with corresponding species, voucher, and GenBank information.
**Table S2** Results of all DIP analyses.
**Table S3** Significant f‐branch metrics.
Please note: Wiley is not responsible for the content or functionality of any Supporting Information supplied by the authors. Any queries (other than missing material) should be directed to the *New Phytologist* Central Office.

## Data Availability

No new data were generated for these analyses. The data used in this study, along with sample sources and collecting permit information, are available in Vargas *et al*. (2020). Analysis scripts and additional materials are accessible on Bitbucket at https://bitbucket.org/kateuckele/costus_introgression/src/master/.
