# Supplementary material for: Prezygotic barriers effectively limit hybridization in a rapid evolutionary radiation
Source: New Phytol. 2024 Oct 14;244(6):2548–60. doi: 10.1111/nph.20187 (PMC11579434; doi:10.1111/nph.20187)
Supplement: Supplementary file 1 — Fig. S1 Network scores for SNaQ analyses. Fig. S2 Phylogenetic networks for clade 1 inferred with SNaQ. Fig. S3 Phylogenetic networks for grade 2 inferred with SNaQ. Fig. S4 Phylogenetic networks for clade 3 inferred with SNaQ. Fig. S5 Phylogenetic networks for subset 4 inferred with SNaQ. Fig. S6 Network scores for PhyloNet analyses. Fig. S7 Phylogenetic networks for clade 1 inferred with PhyloNet. Fig. S8 Phylogenetic networks for grade 2 inferred with PhyloNet. Fig. S9 Phylogenetic networks for clade 3 inferred with PhyloNet. Fig. S10 Phylogenetic networks for subset 4 inferred with PhyloNet. Notes S1 Site pattern frequencies in significant vs nonsignificant D‐statistic tests. Notes S2 DIP analyses to detect and polarize asymmetric introgression. Notes S3 Pseudolikelihood phylogenetic network inference with PhyloNet. Table S1 List of samples with corresponding species, voucher, and GenBank information. Table S2 Results of all DIP analyses. Table S3 Significant f‐branch metrics. Please note: Wiley is not responsible for the content or functionality of any Supporting Information supplied by the authors. Any queries (other than missing material) should be directed to the New Phytologist Central Office. [file NPH-244-2548-s001.pdf]

***New Phytologist* Supporting Information**

Article title: Prezygotic barriers effectively limit hybridization in a rapid evolutionary radiation

Authors: Kathryn A. Uckele, Oscar M. Vargas, Kathleen M. Kay

Article acceptance date: 22 September 2024

The following Supporting Information is available for this article:

Fig. S1 Network scores for SNaQ analyses

Fig. S2 Phylogenetic networks for clade 1 inferred with SNaQ

Fig. S3 Phylogenetic networks for grade 2 inferred with SNaQ

Fig. S4 Phylogenetic networks for clade 3 inferred with SNaQ

Fig. S5 Phylogenetic networks for subset 4 inferred with SNaQ

Fig. S6 Network scores for PhyloNet analyses

Fig. S7 Phylogenetic networks for clade 1 inferred with PhyloNet

Fig. S8 Phylogenetic networks for grade 2 inferred with PhyloNet

Fig. S9 Phylogenetic networks for clade 3 inferred with PhyloNet

Fig. S10 Phylogenetic networks for subset 4 inferred with PhyloNet

Table S1 List of samples with corresponding species, voucher, and GenBank information

Table S2 Results of all DIP analyses

Table S3 Significant f-branch metrics

Notes S1 Site pattern frequencies in significant vs. non-significant D-statistic tests

Notes S2 DIP analyses to detect and polarize asymmetric introgression

Notes S3 Pseudo-likelihood phylogenetic network inference with `PhyloNet`

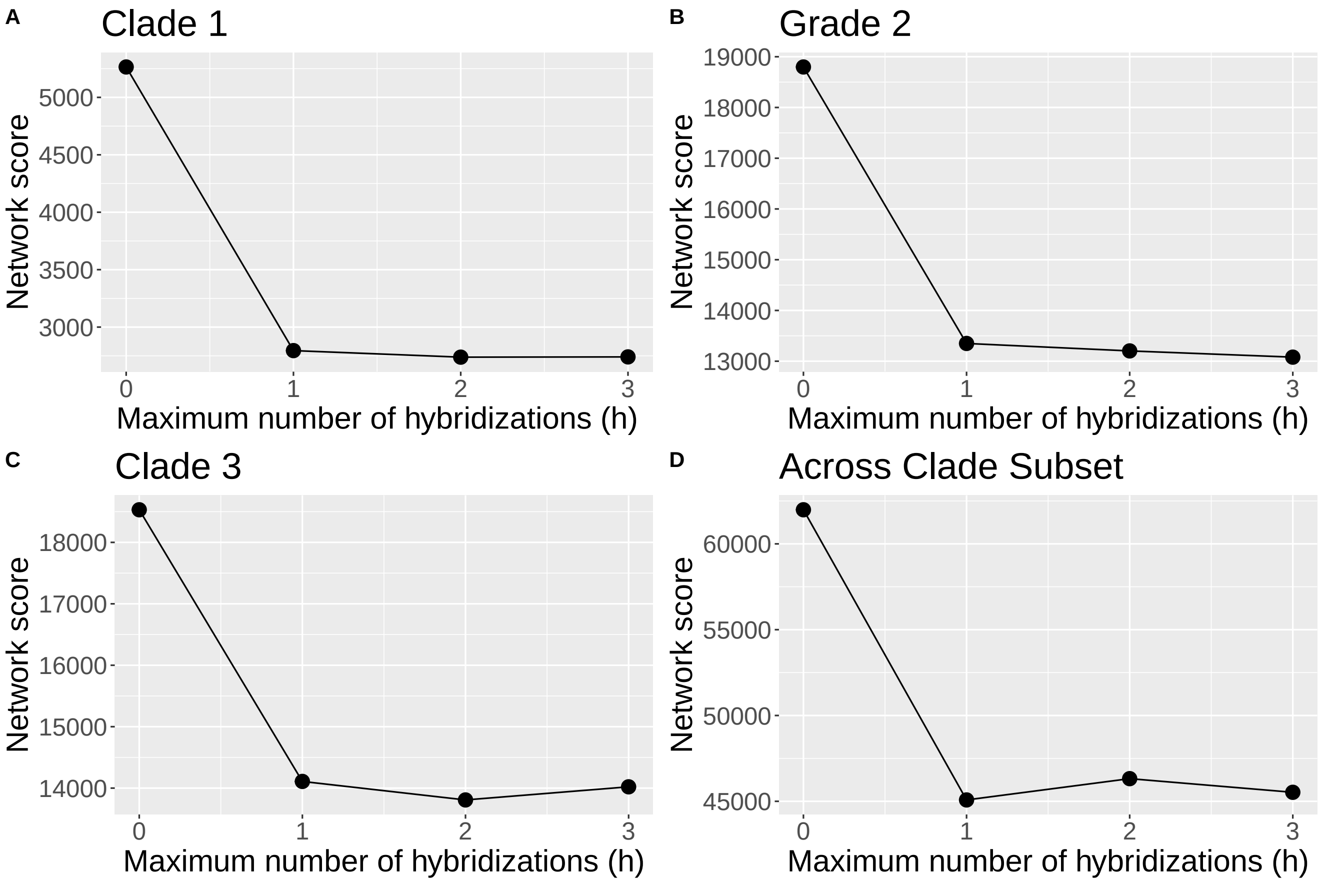

Fig. S1. Network scores (negative log-pseudolikelihood) are plotted for all values of h for each subset of taxa analyzed with `SNaQ` (A – D). To estimate the number of hybridization events for each subset, we utilized a slope heuristic approach (described in [Solís-Lemus & Ané, 2016]). For each subset, the sharpest decrease in network scores was from $h = 0$ to $h = 1$, indicating that one hybridization event ($h = 1$) is best supported by the data.

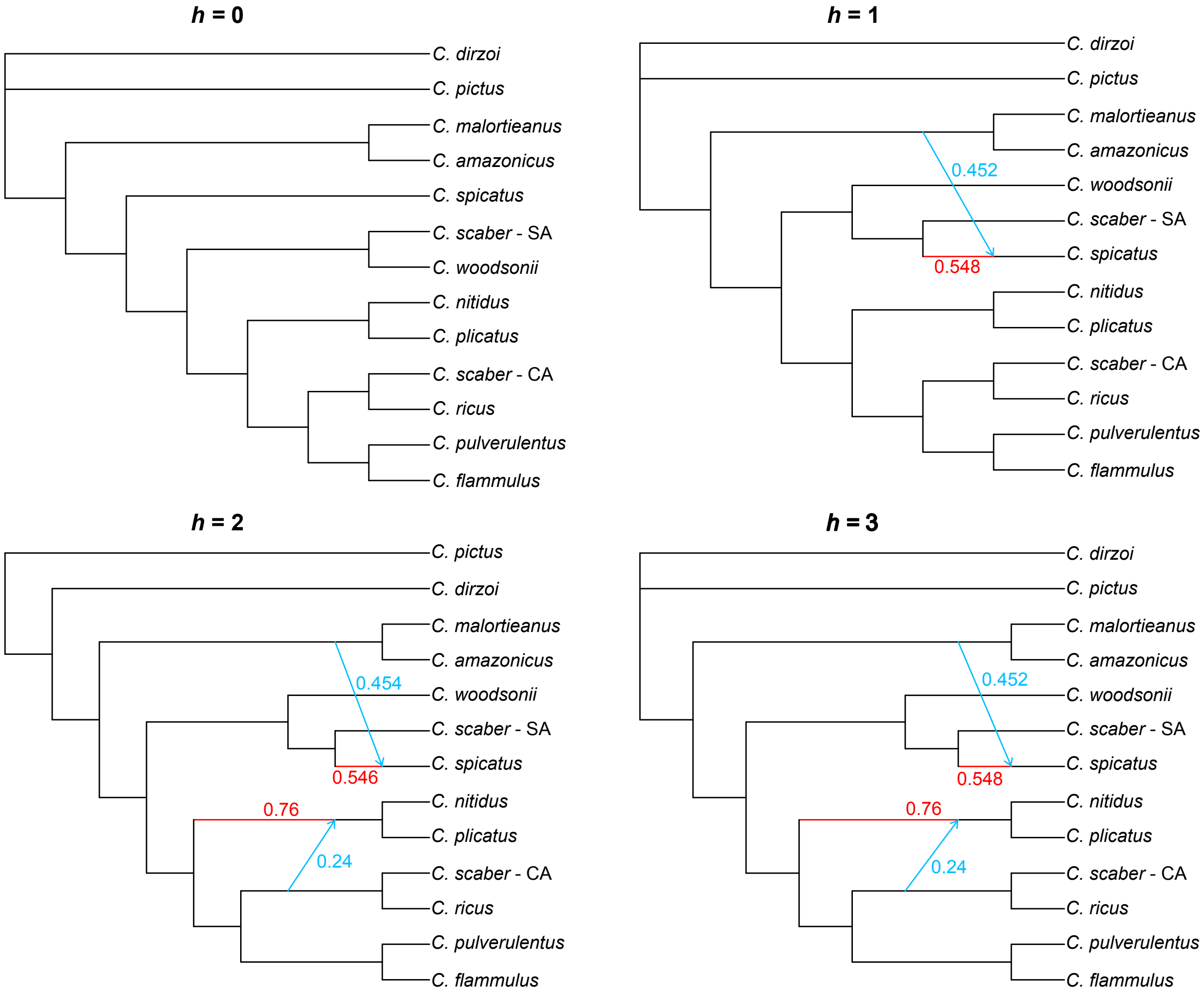

Fig. S2. Phylogenetic network inference with SNaQ for the first taxon subset that form a clade of predominantly Central American *Costus* species. We ran four models, each with a different number of hybridization events from none to three ($h = 0$–3). Twenty independent runs were conducted for each model, and the run with the highest pseudolikelihood is shown. Black edges: major tree depicting species relationships. Blue arrows: minor hybrid edges, annotated by their estimated γ. Red edges: major hybrid edges, annotated by $1 - \gamma$.

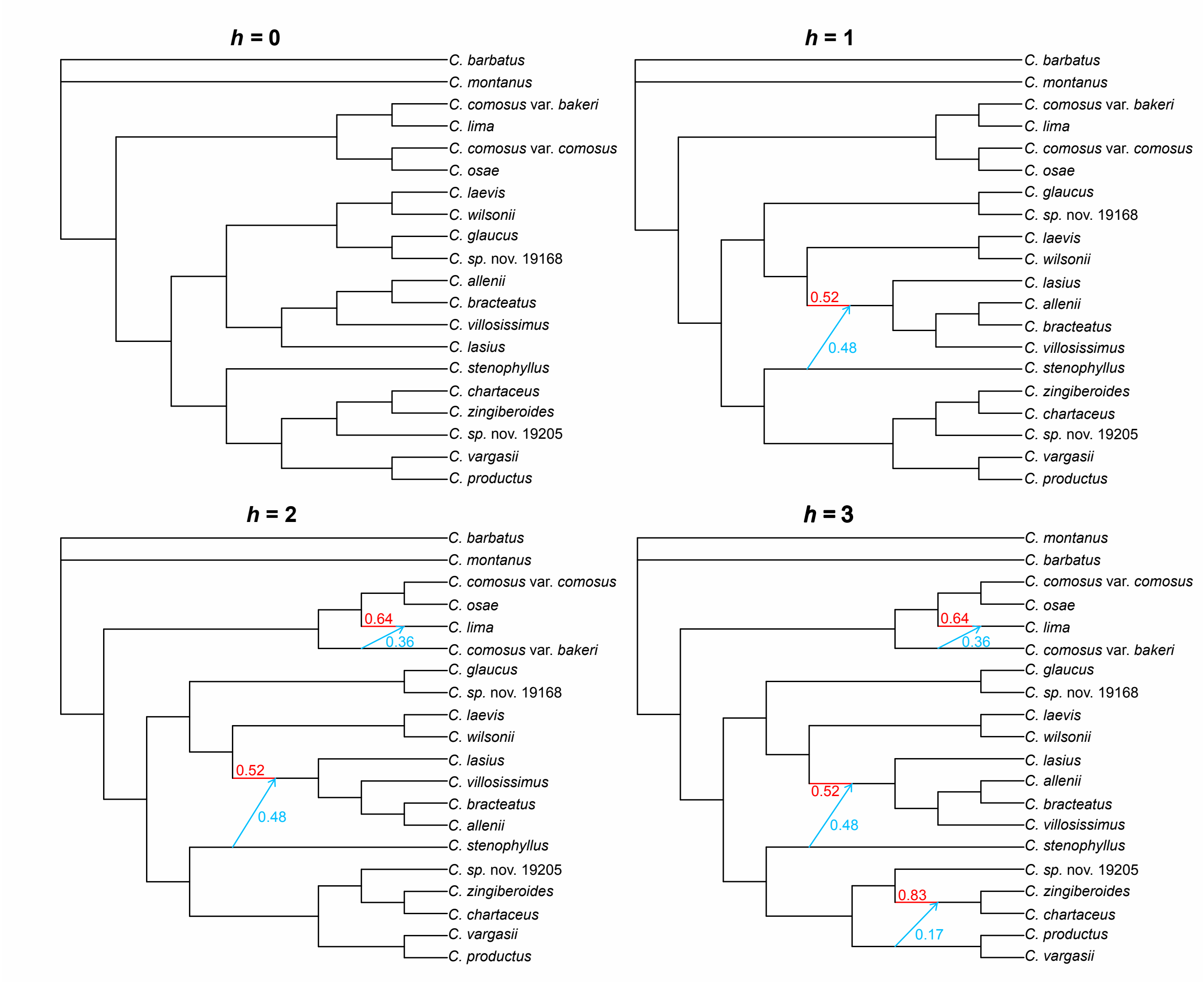

Fig. S3. Phylogenetic network inference with SNaQ for the second taxon subset that form a grade between the first and third taxon subsets. We ran four models, each with a different number of hybridization events from none to three (h = 0–3). Twenty independent runs were conducted for each model, and the run with the highest pseudolikelihood is shown. Black edges: major tree depicting species relationships. Blue arrows: minor hybrid edges, annotated by their estimated γ. Red edges: major hybrid edges, annotated by $1 - \gamma$

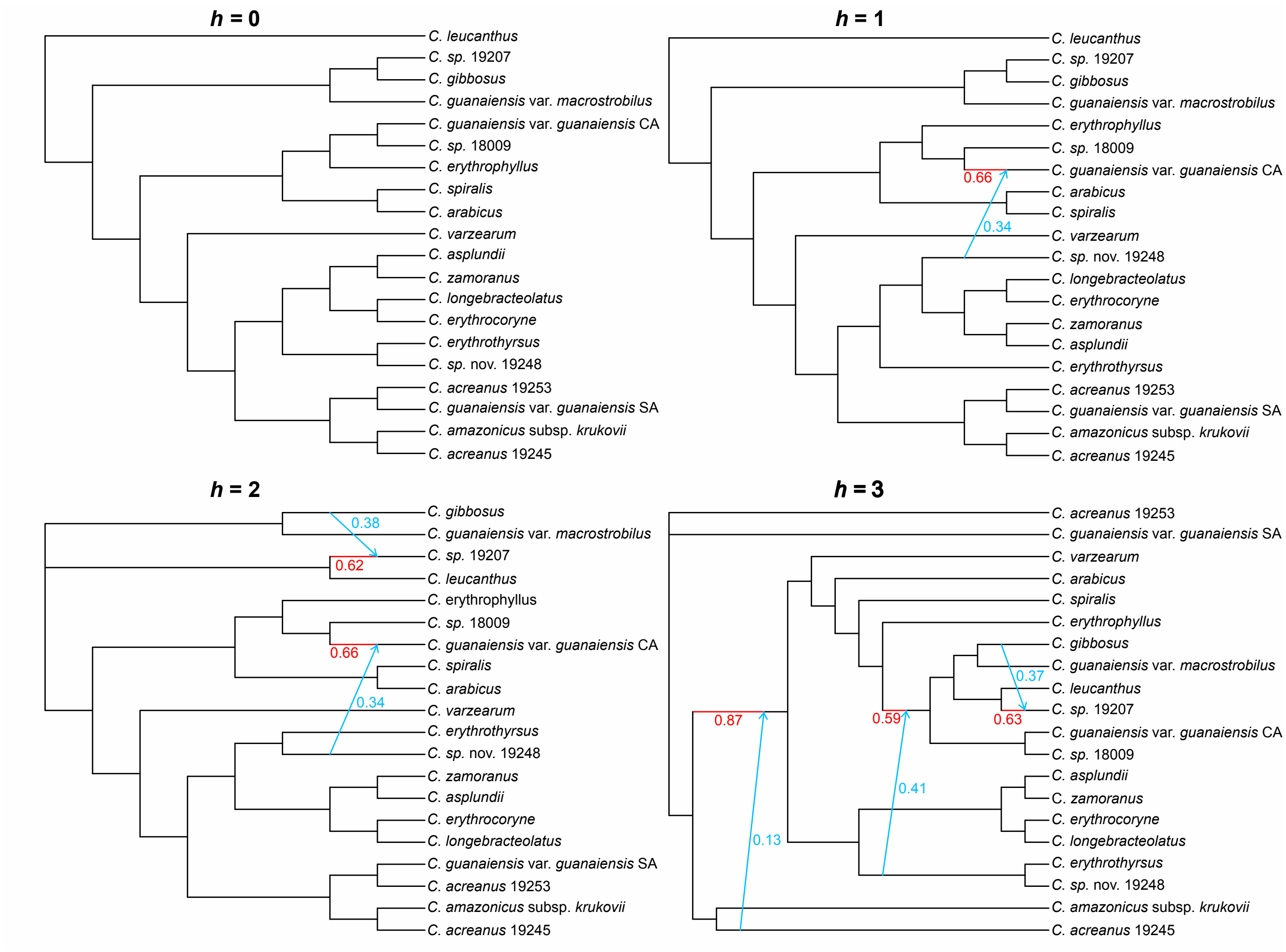

Fig. S4. Phylogenetic network inference with SNaQ for the third taxon subset that includes the members of the Amazonian clade. We ran four models, each with a different number of hybridization events from none to three ($h = 0$–3). Twenty independent runs were conducted for each model, and the run with the highest pseudolikelihood is shown. Black edges: major tree depicting species relationships. Blue arrows: minor hybrid edges, annotated by their estimated γ. Red edges: major hybrid edges, annotated by $1 - \gamma$

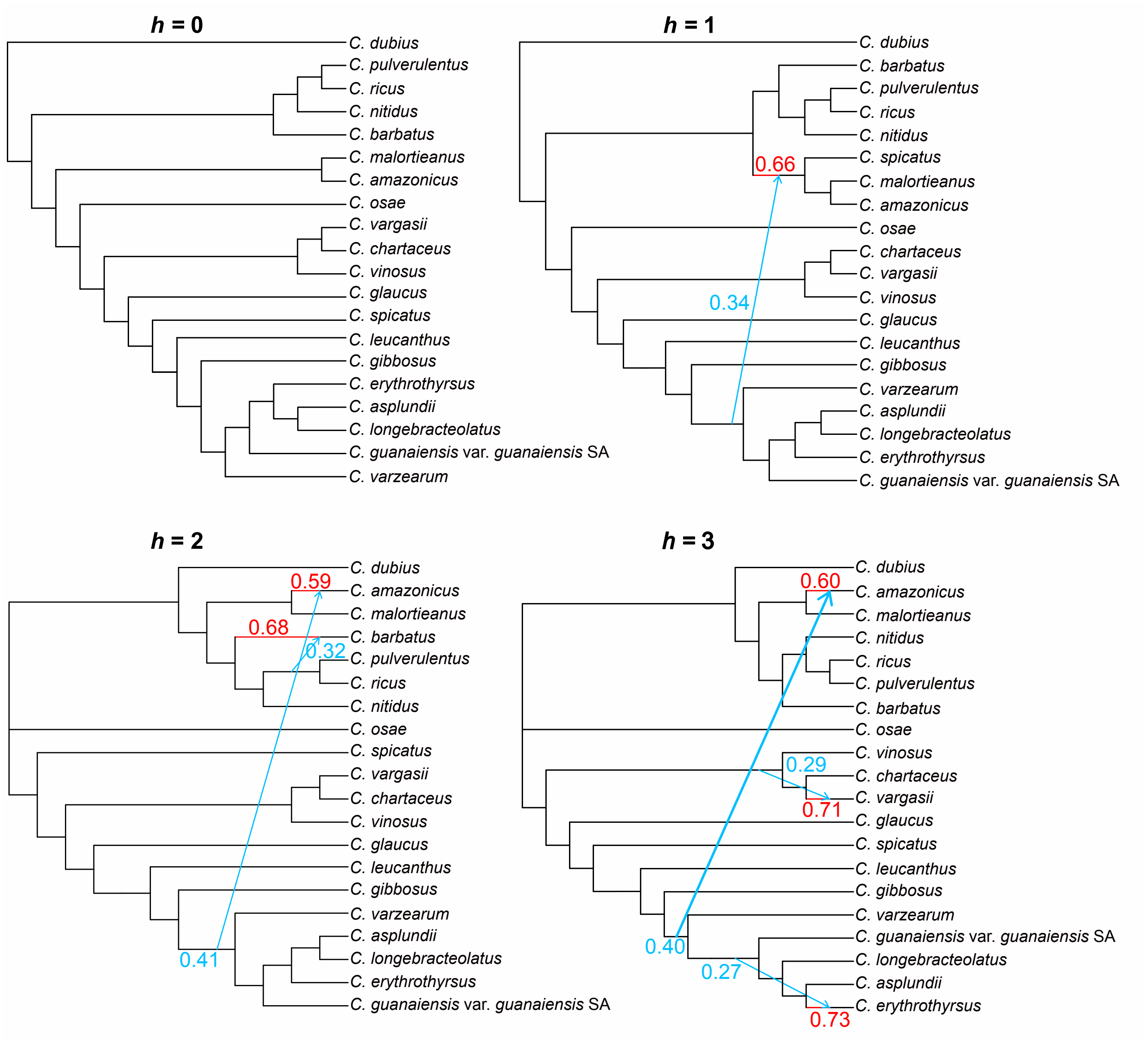

Fig. S5. Phylogenetic network inference with SNaQ for the fourth subset that includes taxa from across the American *Costus* phylogeny. We ran four models, each with a different number of hybridization events from none to three (h = 0–3). Twenty independent runs were conducted for each model, and the run with the highest pseudolikelihood is shown. Black edges: major tree depicting species relationships. Blue arrows: minor hybrid edges, annotated by their estimated γ. Red edges: major hybrid edges, annotated by $1 - \gamma$

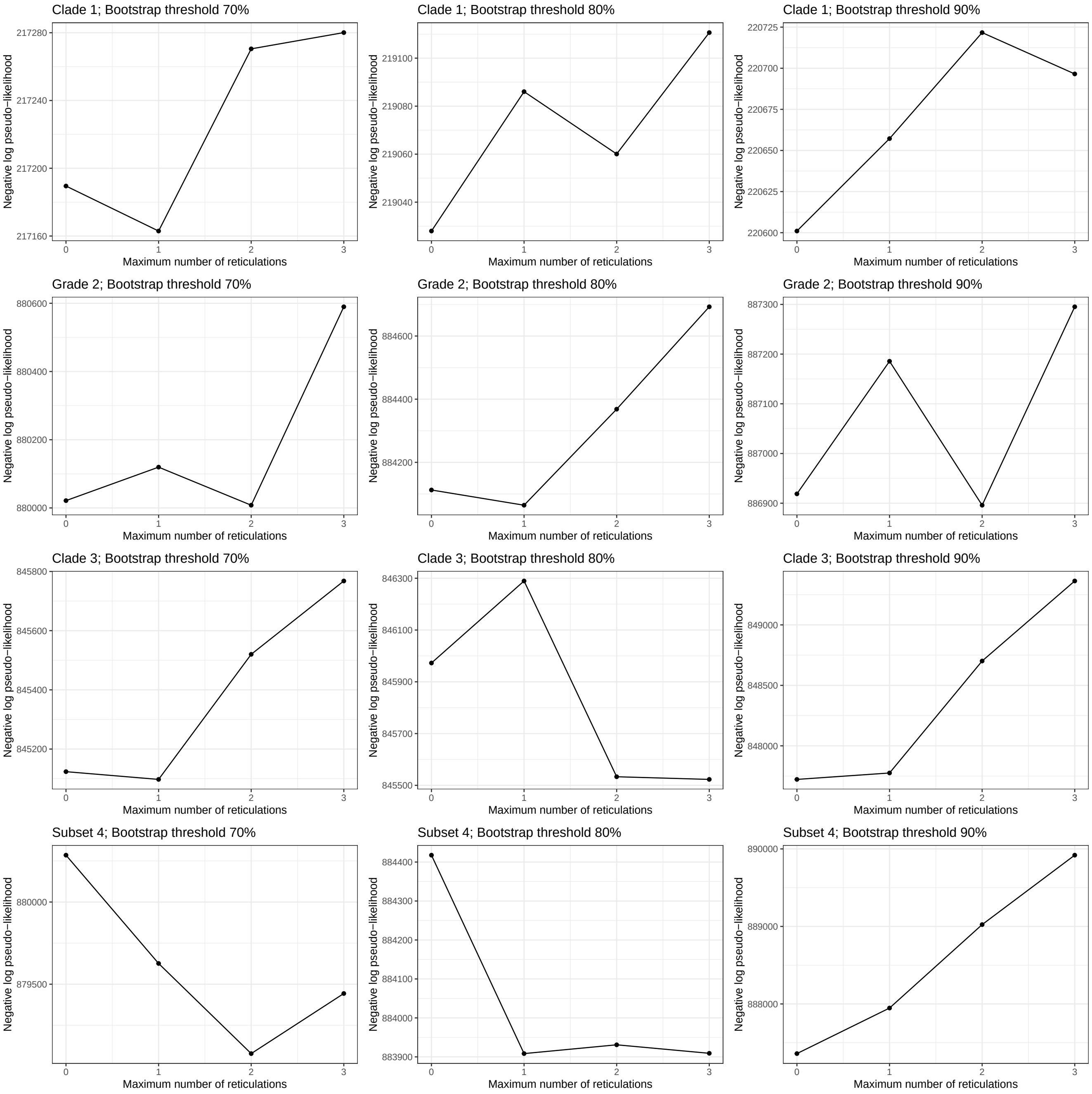

Fig. S6. Negative log-likelihood scores for all `PhyloNet` maximum pseudo-likelihood inferences. For each taxon subset and bootstrap threshold, four inferences were conducted with 0, 1, 2, and 3 maximum number of reticulations. A slope heuristic approach [Solís-Lemus & Ané, 2016] was used to determine the best number of reticulations.

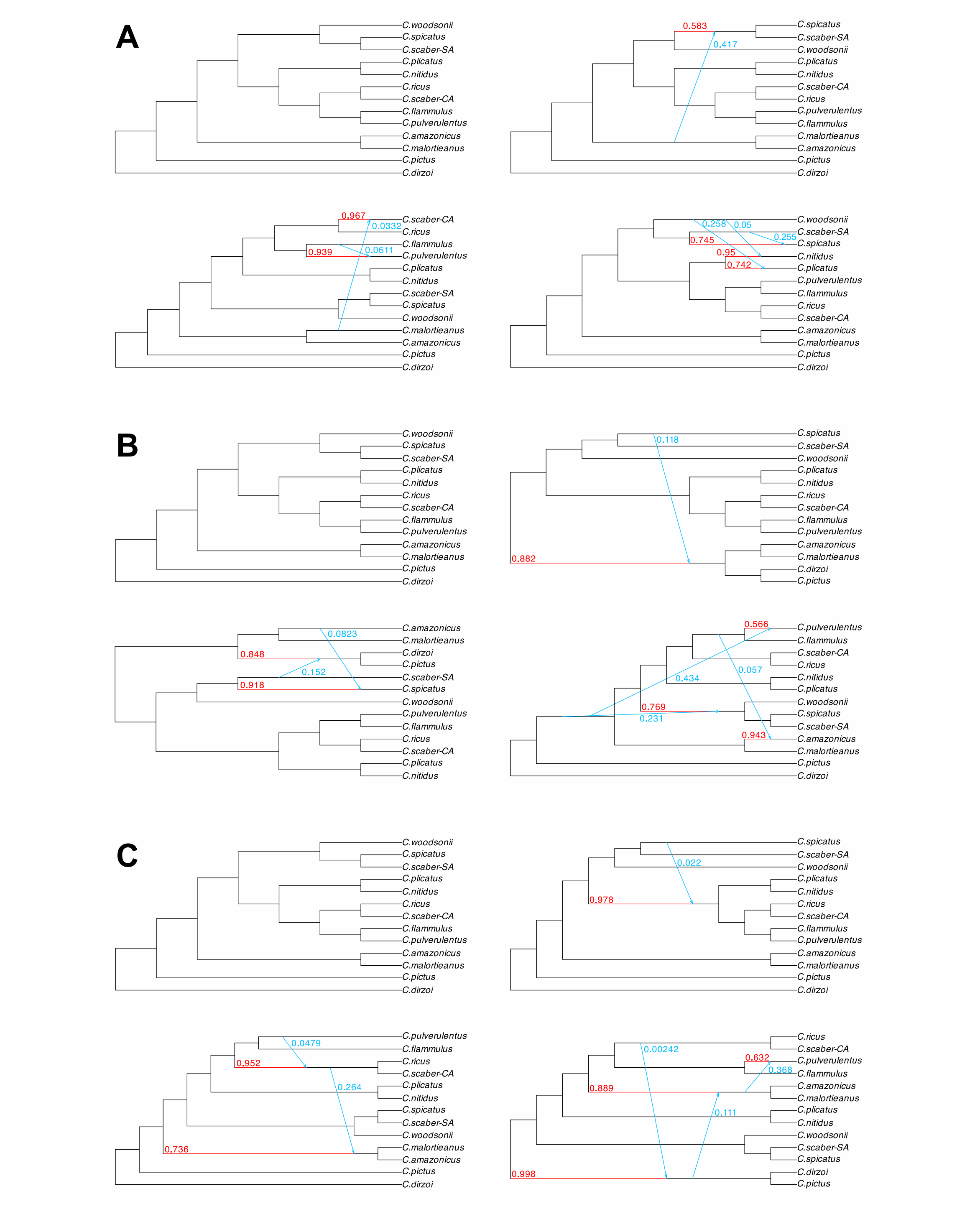

Fig. S7. Phylogenetic networks for clade 1 were inferred with the `PhyloNet` maximum pseudo-likelihood inference method. Poorly supported edges were contracted according to three bootstrap thresholds, 70, 80, and 90%, shown in panels A, B, and C, respectively. For each bootstrap threshold, inferences were conducted with 0–3 maximum number of reticulations.

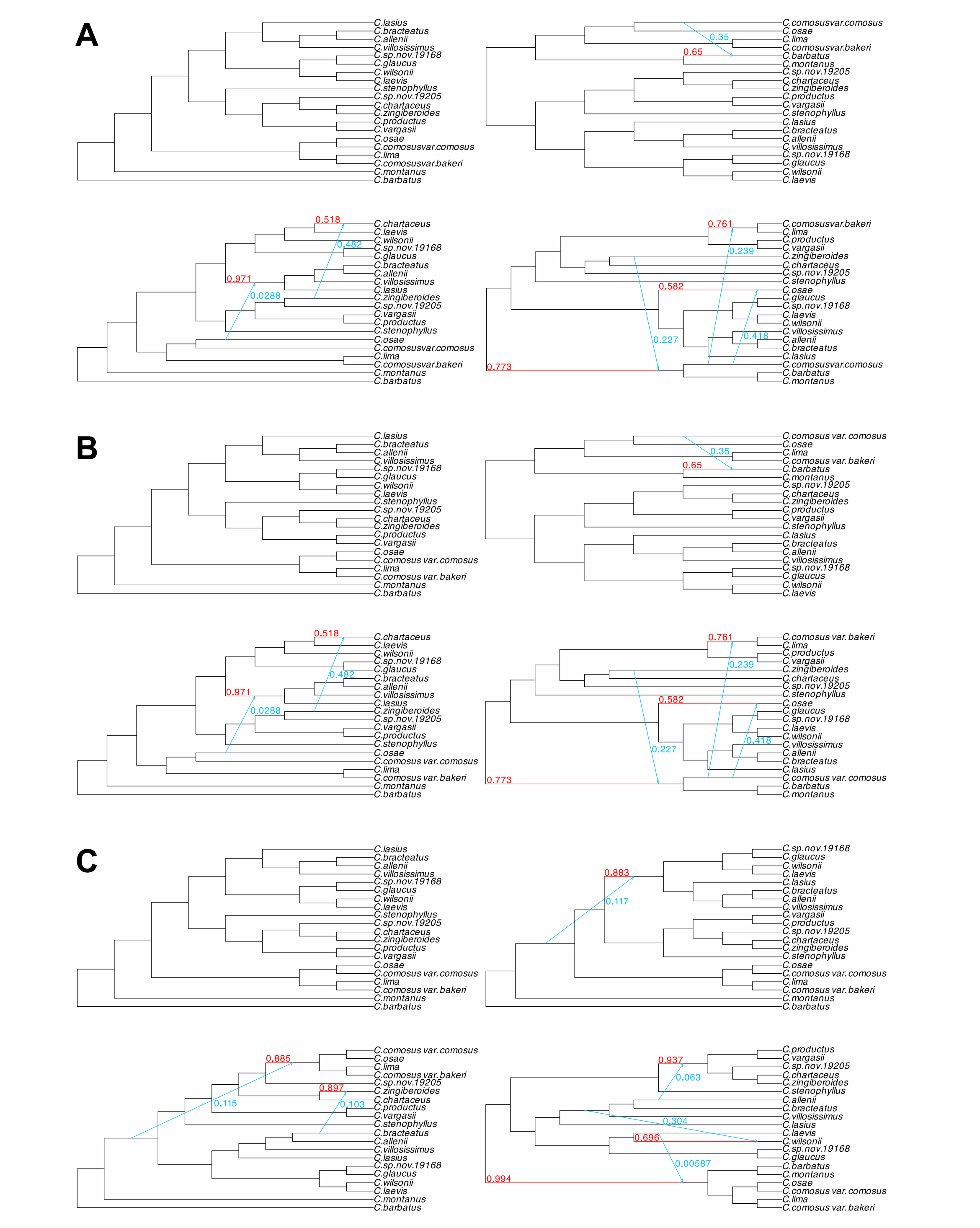

Fig. S8. Phylogenetic networks for grade 2 were inferred with the `PhyloNet` maximum pseudo-likelihood inference method. Poorly supported edges were contracted according to three bootstrap thresholds, 70, 80, and 90%, shown in panels A, B, and C, respectively. For each bootstrap threshold, inferences were conducted with 0–3 maximum number of reticulations.

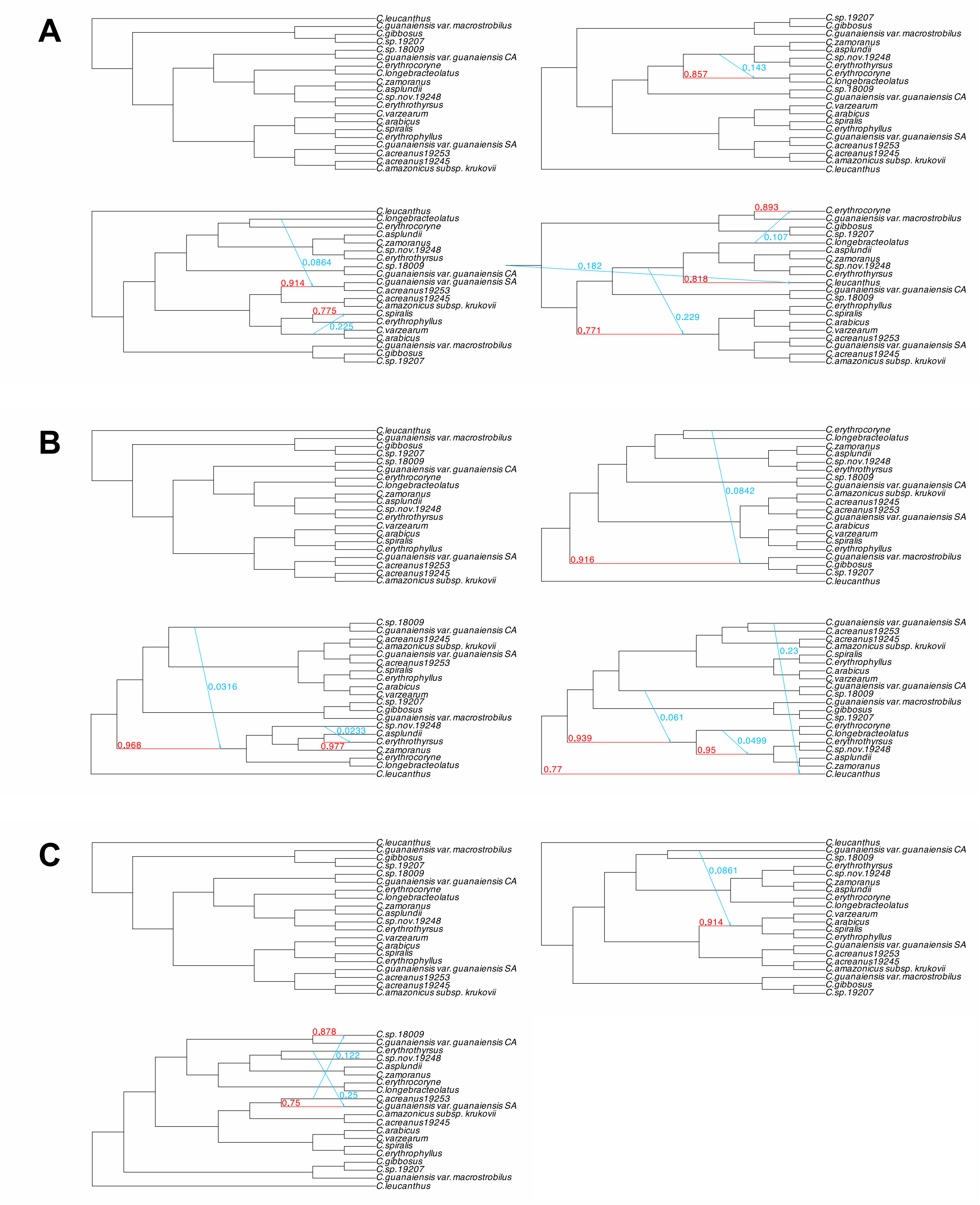

Fig. S9. Phylogenetic networks for clade 3 were inferred with the `PhyloNet` maximum pseudo-likelihood inference method. Poorly supported edges were contracted according to three bootstrap thresholds, 70, 80, and 90%, shown in panels A, B, and C, respectively. For each bootstrap threshold, inferences were conducted with 0–3 maximum number of reticulations. The phylogenetic network inferred using gene trees contracted under a 90% bootstrap threshold with three reticulation is not shown due to its topological complexity and poor support relative to the backbone phylogeny without reticulations (Fig. S8).

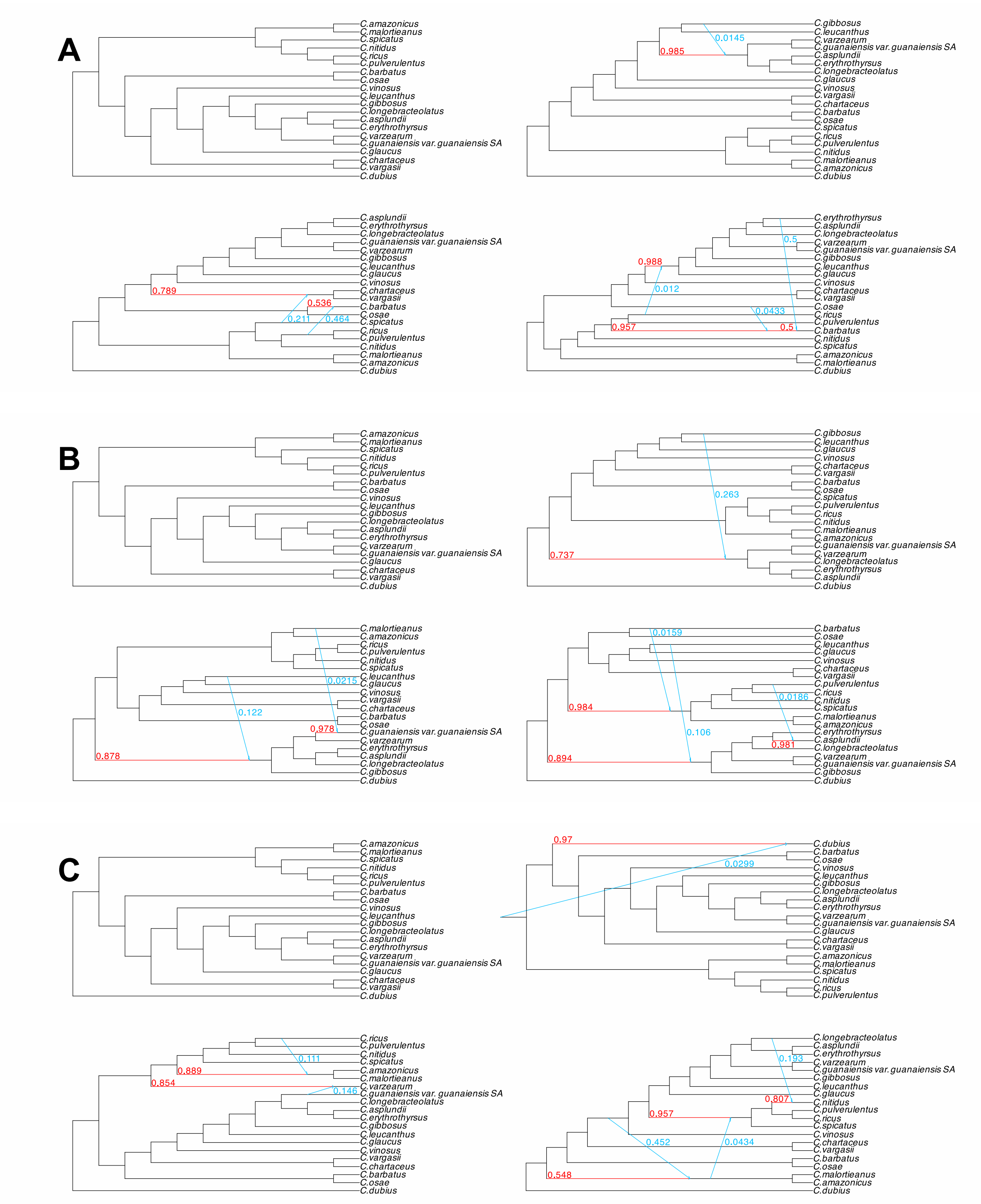

Fig. S10. Phylogenetic networks for subset 4 were inferred with the `PhyloNet` maximum pseudo-likelihood inference method. Poorly supported edges were contracted according to three bootstrap thresholds, 70, 80, and 90%, shown in panels A, B, and C, respectively. For each bootstrap threshold, inferences were conducted with 0–3 maximum number of reticulations.

Table S1. *Costus* samples included in clade-wide phylogenomic analyses of introgression. When more than one sample was available per taxa, the sample indicated by an asterisk was utilized for RT test and `SNaQ` analyses. All samples were utilized in the `Dsuite` analyses. The rightmost column provides the original species names used in Vargas *et al.* [2020]. HUPCH: Herbario de la Universidad Peruana Cayetano Heredia, JBNB: Jardin Botanique National de Belgique, MICH: University of Michigan Herbarium, MO: Missouri Botanical Garden Herbarium, MSC: Michigan State University Herbarium, NTBG: National Tropical Botanical Garden, TWC: Tom Wood personal collection, UBG: Utrecht Botanic Gardens, UC: Herbarium University of California - Berkeley, UCB: Botanical Garden University of California - Berkeley, UCONN: Greenhouses University of Connecticut, UCSC: Greenhouses University of California - Santa Cruz, US: United States National Herbarium, BO: Bolivia, BR: Brazil, CM: Cameroon, CO: Colombia, CR: Costa Rica, DM: Dominica, EC: Ecuador, FG: French Guiana, GY: Guyana, MX: Mexico, PA: Panama, PE: Peru.

Species	Voucher	Collected	Country	GenBank BioSample	Vargas et al. 2020
C. acreanus 19245	Skinner R3265	UC	PE	SAMN13811711	*C. acreanus* 19245
C. acreanus 19253 (Loes.) Maas	Rimanchi 10995	US	PE	SAMN13811712	*C. acreanus* 19253
C. allenii Maas	Kay 0314	MSC	PA	SAMN13811723	*C. allenii* 98050
C. amazonicus (Loes.) J.F.Macbr.	Skinner R3198	UCSC	EC	SAMN13811725	*C. amazonicus* 19020
C. amazonicus subsp. *krukovii* Maas	1972GR00353, Prance 12826	UBG	BR	SAMN13811726	*C. amazonicus* subsp. *krukovii* 98072
C. arabicus L.	1995GRO1263, Maas s.n.	UBG	BR	SAMN13811727	*C. arabicus* 98080
C. asplundii (Maas) Maas	Maguiña 336	HUPCH	PE	SAMN13811728	*C. asplundii* 18058

Table S1 continued from previous page

Species	Voucher	Collected	Country	GB Bio Sample	Vargas et al. 2020
C. barbatus Suess.	Skinner R3213	NTBG	CR	SAMN13811729	*C. barbatus* 19242
C. bracteatus Rowlee	Harenčár 18-005 (EA0041)	UC	CR	SAMN13811731	*C. bracteatus* 18030
C. chartaceus Maas	Kress 90-3124	UCONN	CO	SAMN13811732	*C. chartaceus* 19269
C. comosus var. *bakeri* (K.Schum.) Maas	Skinner R3315	NTBG	MX	SAMN13811736	*C. comosus* var. *bakeri* 19114
C. comosus var. *comosus* (Jacq.) Roscoe	Schemske 032 (T. Wood living coll.)	MSC		SAMN13811737	*C. comosus* var. *comosus* 98173
C. dirzoi García-Mend. & G.Ibarra	Ibarra 996	MO	MX	SAMN13811738	*C. dirzoi* 19085
**C. dirzoi* García-Mend. & G.Ibarra	1980GR00128, Rooden 812	UBG	MX	SAMN13811739	*C. dirzoi* 98079
C. erythrocoryne K.Schum.	1994GR02117, Maas s.n.	UBG	PE	SAMN13811743	*C. erythrocoryne* 98073
C. erythrophyllus Loes.	Skinner R3306	NTBG	CO	SAMN13811714	*C. erythrophyllus* 19206
C. erythrophyllus Loes.	Kress 94-5379	US		SAMN13811734	*C. erythrophyllus* 19256

Table S1 continued from previous page

Species	Voucher	Collected	Country	GB Bio Sample	Vargas et al. 2020
**C. erythrophyllus* Loes.	Kay 038	MSC		SAMN13811744	*C. erythrophyllus* 98168
C. erythrothyrsus	Kay 0339	MSC		SAMN13811745	*C. erythrothyrsus* 98174
C. flammulus Juárez	Kay 18-001(KK0058)	UC	CR	SAMN13811721	*C. sp.* nov. 18020
**C. flammulus* Juárez	Kay 18-001(KK0073)	UC	CR	SAMN13811722	*C. sp.* nov. 18049
C. glaucus Maas	Skinner R3364	NTBG	CR	SAMN13811746	*C. glaucus* 19166
**C. glaucus* Maas	1974GR00415, Mass 1462	UBG	CR	SAMN13811747	*C. glaucus* 98077
C. guanaiensis var. *guanaiensis* CA Rusby	Skinner R3140	NTBG	PA	SAMN13811753	*C. guanaiensis* var. *guanaiensis* CA 19203
C. guanaiensis var. *guanaiensis* SA Rusby	Maguiña 326	HUPCH	PE	SAMN13811749	*C. guanaiensis* var. *guanaiensis* SA 18056
**C. guanaiensis* var. *guanaiensis* SA Rusby	Maguiña 338	HUPCH	PE	SAMN13811750	*C. guanaiensis* var. *guanaiensis* SA 18059
C. guanaiensis var. *macrostrobilus* (K.Schum.) Maas	Kay 0319 (UCSC-GH 41)	MSC	PA	SAMN13811751	*C. guanaiensis* var. *macrostrobilus* 18077

Table S1 continued from previous page

Species	Voucher	Collected	Country	GB Bio Sample	Vargas et al. 2020
C. gibbosus D.Skinner & Maas	Skinner s.n.	UCSC	BR	SAMN13811748	*C. guanaiensis* var. *tarmicus* 18002
**C. gibbosus* D.Skinner & Maas	Skinner R3323	NTBG	EC	SAMN13811717	*C. guanaiensis* var. *tarmicus* 19243
C. laevis Ruiz & Pav.	Harenčár 18-005 (JH0034)	UC	CR	SAMN13811730	*C. laevis* 18029
C. laevis Ruiz & Pav.	Kay 0320	MSC	CR	SAMN13811754	*C. laevis* 18031
**C. laevis* Ruiz & Pav.	Harenčár 18-010 (JH0071)	UC	CR	SAMN13811755	*C. laevis* 18032
**C. laevis* Ruiz & Pav.	Harenčár 18-016 (EA0087)	UC	CR	SAMN13811756	*C. laevis* 19191
C. laevis Ruiz & Pav.	Avila 0068 (EA0070)	UC	CR	SAMN13811792	*C. laevis* 19218
C. laevis Ruiz & Pav.	Kay 0310 (UCSC-GH 54)	MSC	PA	SAMN13811757	*C. laevis* RD299
**C. lasius* Loes.	Kay 0321 (UCSC-GH 125)	MSC	PA	SAMN13811758	*C. lasius* 18080
C. lasius Loes.	Plowman 11668	NTBG	PE	SAMN13811759	*C. lasius* 19117
C. leucanthus Maas	86GR00130, Maas 6527	UBG	CO	SAMN13811760	*C. leucanthus* 98075
C. lima K.Schum.	Kay 023 (DG0028)	MSC	CR	SAMN13811761	*C. lima* 18035

Table S1 continued from previous page

Species	Voucher	Collected	Country	GB Bio Sample	Vargas et al. 2020
C. lima K.Schum.	Kay 023 (DG0025)	MSC	CR	SAMN13811762	*C. lima* 19228
**C. lima* K.Schum.	75-0400	JBNB	CO	SAMN13811763	*C. lima* 98165
C. longebracteolatus Maas	Maguiña 344	HUPCH	PE	SAMN13811764	*C. longebracteolatus* 18061
**C. longebracteolatus* Maas	s. n.	TWC	CR	SAMN13811765	*C. longebracteolatus* 98176
C. lucanusianus J.Braun & K.Schum.	1968GR00220	UBG	CM	SAMN13811766	*C. lucanusianus* 99103
**C. malortieanus* H.Wendl.	Kay 0322 (RM0011)	MSC	CR	SAMN13811767	*C. malortieanus* 18036
C. malortieanus H.Wendl.	Kay 0322 (JH0028)	MSC	CR	SAMN13811768	*C. malortieanus* 19216
C. montanus Maas	Kay 18-002 (JF0001)	UC	CR	SAMN13811769	*C. montanus* 18037
**C. montanus* Maas	Kay 18-002 (KK0075)	UC	CR	SAMN13811770	*C. montanus* 19217
**C. nitidus* Maas	Skinner R3088	UCSC	CR	SAMN13811771	*C. nitidus* 18001
C. osae Maas & H.Maas	Harenčár 18-009 (JH0096)	UC	CR	SAMN13811772	*C. osae* 18038

Table S1 continued from previous page

Species	Voucher	Collected	Country	GB Bio Sample	Vargas et al. 2020
**C. osae* Maas & H.Maas	Harenčár 18-009 (JH0081)	UC	CR	SAMN13811773	*C. osae* 19005
C. pictus D.Don	Norris 13289	MICH	MX	SAMN13811775	*C. pictus* 19284
**C. pictus* D.Don	00-5272	JBNB		SAMN13811776	*C. pictus* 98160
**C. plicatus* Maas	Grossenbacher 0026	UC	CR	SAMN13811777	*C. plicatus* 18039
C. plicatus Maas	Grossenbacher 0026 (PJ0018)	UC	CR	SAMN13811778	*C. plicatus* 19067
C. productus Gleason ex Maas	Kress 94-3708	US		SAMN13811779	*C. productus* 19263
C. productus Gleason ex Maas	Skinner s.n.	UCONN		SAMN13811780	*C. productus* 19281
**C. productus* Gleason ex Maas	Kay 039 (T. Wood living coll. (labeled C. curvibracteatus))	MSC		SAMN13811781	*C. productus* 98170
C. pulverulentus C.Presl	Kay 0326	MSC	CR	SAMN13811782	*C. pulverulentus* 18040
C. pulverulentus C.Presl	Harenčár 18-004 (EA0030)	UC	CR	SAMN13811783	*C. pulverulentus* 18041
**C. pulverulentus* C.Presl	Harenčár 18-012 (PG0017)	UC	CR	SAMN13811784	*C. pulverulentus* 18042
C. pulverulentus C.Presl	Kay 022 (UCSC-GH 133)	MSC	CR	SAMN13811785	*C. pulverulentus* 18081

Table S1 continued from previous page

Species	Voucher	Collected	Country	GB Bio Sample	Vargas et al. 2020
C. pulverulentus C.Presl	Kay 0328 (UCSC-GH 151)	MSC	PA	SAMN13811788	*C. pulverulentus* 18084
C. pulverulentus C.Presl	Skinner R3304	NTBG	CO	SAMN13811789	*C. pulverulentus* 19119
C. ricus Maas & H.Maas	Avila 0068 (JH0075)	UC	CR	SAMN13811791	*C. ricus* 18043
**C. scaber* CA Ruiz & Pav.	not collected (la Gamba, Finca la Virgen)	NA	CR	SAMN13811719	*C. scaber* 18044
C. scaber CA Ruiz & Pav.	Kay 0330	MSC	CR	SAMN13811794	*C. scaber* 18045
C. scaber CA Ruiz & Pav.	Harenčár 18-008 (PJ0004)	UC	CR	SAMN13811795	*C. scaber* 18046
C. scaber CA Ruiz & Pav.	Kay 021 (UCSC-GH 170)	MSC	CR	SAMN13811799	*C. scaber* 18087
C. scaber CA Ruiz & Pav.	Kay 0325 (UCSC-GH 109)	MSC	PA	SAMN13811796	*C. scaber* 18078
**C. scaber* SA Ruiz & Pav.	Kay 0329 (UCSC-GH 160)	MSC	BO	SAMN13811797	*C. scaber* 18085
C. scaber SA Ruiz & Pav.	Maguiña 301	HUPCH	PE	SAMN13811793	*C. scaber* 18025
C. scaber SA Ruiz & Pav.	Maguiña 339	HUPCH	PE	SAMN13811720	*C. scaber* 18060
C. scaber SA Ruiz & Pav.	Kay 032 (UCSC-GH 162)	MSC	BO	SAMN13811798	*C. scaber* 18086

Table S1 continued from previous page

Species	Voucher	Collected	Country	GB Bio Sample	Vargas et al. 2020
C. scaber SA Ruiz & Pav.	Skinner R3215	NTBG	GY	SAMN13811800	*C. scaber* 19167
C. scaber SA Ruiz & Pav.	Skinner R3220	NTBG	GY	SAMN13811806	*C. sp.* 19122
C. sp. 18009	Skinner R3079	UCSC	EC	SAMN13811724	*C. amazonicus* GUAL 18009
C. sp. nov. 19168	Skinner R3316	NTBG	MX	SAMN13811774	*C. sp.* nov. 19118
**C. sp.* nov. 19168	Skinner R3314	NTBG	MX	SAMN13811718	*C. sp.* nov. 19168
C. sp. nov. 19205	Skinner R3226	NTBG	CO	SAMN13811713	*C. sp.* nov. 19205
C. sp. 19207	Skinner R3200	NTBG	EC	SAMN13811716	*C. sp.* 19207
C. sp. nov. 19248	Skinner R3130	UC	PE	SAMN13811733	*C. sp.* nov. 19248
**C. spicatus* (Jacq.) Sw.	Skinner R3028	UC	DM	SAMN13811801	*C. spicatus* 19251
C. spicatus (Jacq.) Sw.	Kress 02-7143	UCONN	DM	SAMN13811802	*C. spicatus* 19272
C. spiralis	Croat 102665	MO	FG	SAMN13811804	*C. spiralis* var. *villosus* 19077
**C. spiralis*	Skinner R3218	NTBG	GY	SAMN13811805	*C. spiralis* var. *villosus* 19209
C. stenophyllus Standl. & L.O.Williams	Harenčár 18-013 (DG0012)	UC	CR	SAMN13811807	*C. stenophyllus* 18048

Table S1 continued from previous page

Species	Voucher	Collected	Country	GB Bio Sample	Vargas et al. 2020
**C. stenophyllus* Standl. & L.O.Williams	Harenčár 18-013 (DG0008)	UC	CR	SAMN13811808	*C. stenophyllus* 19227
C. vargasii Maas & H.Maas	Skinner R3264	NTBG	PE	SAMN13811809	*C. vargasii* 19210
**C. varzearum* Maas	Skinner R1394	UC		SAMN13811810	*C. varzearum* 19252
C. varzearum Maas	1971GR00153, Prance 12064	UBG	BR	SAMN13811811	*C. varzearum* 98076
**C. villosissimus* Jacq.	Kay 0313 (UCSC-GH 214)	MSC	PA	SAMN13811812	*C. villosissimus* 18090
C. villosissimus Jacq.	Skinner R3074	NTBG	EC	SAMN13811813	*C. villosissimus* 19169
C. villosissimus Jacq.	Kay 0313	MSC	PA	SAMN13811814	*C. villosissimus* 98048
**C. vinosus* Maas	Dressler s.n.	UCONN	PA	SAMN13811815	*C. vinosus* 19273
C. vinosus Maas	TW s.n.	TWC	PA	SAMN13811816	*C. vinosus* 98171
**C. wilsonii* Maas	Harenčár 18-014 (JH0142)	UC	CR	SAMN13811817	*C. wilsonii* 18050
C. wilsonii Maas	Harenčár 18-015 (PJ0038)	UC	CR	SAMN13811818	*C. wilsonii* 18051
**C. woodsonii* Maas	Harenčár 18-007 (JH0051)	UC	CR	SAMN13811819	*C. woodsonii* 18052
C. woodsonii Maas	Kay 036 (UCSC-GH 208)	MSC	PA	SAMN13811820	*C. woodsonii* 18089

Table S1 continued from previous page

Species	Voucher	Collected	Country	GB Bio Sample	Vargas et al. 2020
C. woodsonii Maas	Harenčár 18-007 (JH0069)	UC	CR	SAMN13811821	*C. woodsonii* 19232
C. zamoranus Steyerm.	Skinner R3325	NTBG	EC	SAMN13811822	*C. zamoranus* 19170
C. zingiberoides J.F.Macbr.	86-0010	JBNB	PE	SAMN13811823	*C. zingiberoides* 98162

Table S2. The results of DIP analyses [Forsythe *et al.*, 2020] to polarize the direction of introgression for select pairs of species that exhibited a significant signal of introgression with RT tests.

P1, P2, P3, O	P-value for ΔK_{23}	P-value for ΔK_{12}	P-value for ΔK_{13}	2xDIP direction	2xDIP p-value	3xDIP direction	3xDIP p-value
C. malortieanus, C. amazonicus, C. acreanus 19245, C. lucanusianus	0	0	0.1	P3P2	0.192	P3P2	0.002
C. malortieanus, C. amazonicus, C. acreanus 19253, C. lucanusianus	0.003	0	0.124	P3P2	0.034	P3P2	0
C. malortieanus, C. amazonicus, C. sp. 18009, C. lucanusianus	0	0	0.092	P3P2	0.354	P3P2	0.016
C. malortieanus, C. amazonicus, C. amazonicus subsp. krukovii, C. lucanusianus	0	0	0.091	P3P2	0.318	P3P2	0.01
C. malortieanus, C. amazonicus, C. arabicus, C. lucanusianus	0	0	0.496	P3P2	0.006	P3P2	0
C. malortieanus, C. amazonicus, C. asplundii, C. lucanusianus	0	0	0.057	P3P2	0.51	P3P2	0.026
C. malortieanus, C. amazonicus, C. erythrocoryne, C. lucanusianus	0.022	0	0.392	P3P2	0.112	P3P2	0
C. malortieanus, C. amazonicus, C. erythrophyllus, C. lucanusianus	0	0	0.07	P3P2	0.174	P3P2	0.004
C. malortieanus, C. amazonicus, C. erythrothyrsus, C. lucanusianus	0	0	0.017	P3P2	0.498	P3P2	0.02
C. malortieanus, C. amazonicus, C. guanaiensis var guanaiensis CA, C. lucanusianus	0	0	0.073	P3P2	0.38	P3P2	0.118
C. malortieanus, C. amazonicus, C. guanaiensis var guanaiensis SA, C. lucanusianus	0	0	0.016	P3P2	0.5	P3P2	0.006

Table S2 continued from previous page

P1, P2, P3, O	P-value for ΔK_{23}	P-value for ΔK_{12}	P-value for ΔK_{13}	2xDIP direction	2xDIP p-value	3xDIP direction	3xDIP p-value
C. malortieanus, C. amazonicus, C. guanaiensis var. macrostrobilus, C. lucanusianus	0	0	0.082	P3P2	0.348	P3P2	0.006
C. malortieanus, C. amazonicus, C. gibbosus, C. lucanusianus	0	0	0.057	P3P2	0.316	P3P2	0
C. malortieanus, C. amazonicus, C. leucanthus, C. lucanusianus	0	0.002	0.003	P2P3	0.826	P3P2	0.024
C. malortieanus, C. amazonicus, C. longebracteolatus, C. lucanusianus	0	0	0.024	P3P2	0.798	P3P2	0.174
C. malortieanus, C. amazonicus, C. sp. 19207, C. lucanusianus	0	0	0.062	P3P2	0.292	P3P2	0
C. malortieanus, C. amazonicus, C. sp. nov. 19248, C. lucanusianus	0	0	0.127	P3P2	0.204	P3P2	0
C. malortieanus, C. amazonicus, C. spiralis, C. lucanusianus	0	0	0.163	P3P2	0.174	P3P2	0
C. malortieanus, C. amazonicus, C. varzearum, C. lucanusianus	0	0	0.016	P3P2	0.462	P3P2	0.014
C. malortieanus, C. amazonicus, C. zamoranus, C. lucanusianus	0.001	0	0.376	P3P2	0.028	P3P2	0
C. spicatus, C. scaber SA, C. nitidus, C. lucanusianus	0.17	0.004	0.458	P3P2	0.196	P3P2	0.61
C. spicatus, C. scaber SA, C. plicatus, C. lucanusianus	0.036	0.005	0.308	P3P2	0.302	P3P2	0.786
C. spicatus, C. scaber SA, C. pulverulentus, C. lucanusianus	0.024	0.001	0.196	P3P2	0.3	P3P2	0.794
C. spicatus, C. scaber SA, C. ricus, C. lucanusianus	0.036	0.016	0.345	P3P2	0.392	P2P3	0.69

Table S2 continued from previous page

P1, P2, P3, O	P-value for ΔK_{23}	P-value for ΔK_{12}	P-value for ΔK_{13}	2xDIP direction	2xDIP p-value	3xDIP direction	3xDIP p-value
C. spicatus, C. scaber SA, C. scaber CA, C. lucanusianus	0	0.004	0.246	P3P2	0.374	P3P2	0.996
C. spicatus, C. scaber SA, C. flammulus, C. lucanusianus	0.005	0.001	0.081	P3P2	0.574	P2P3	0.786
C. spicatus, C. scaber SA, C. woodsonii, C. lucanusianus	0	0	0.384	P3P2	0.09	P3P2	0.658
C. pulverulentus, C. flammulus, C. montanus, C. lucanusianus	0.006	0	0.744	P3P2	0	P3P2	0.002
C. barbatus, C. montanus, C. flammulus, C. lucanusianus	0.001	0.023	0.351	P3P2	0.45	P3P2	0.278
C. guanaiensis var guanaiensis CA, C. sp. 18009, C. erythrophyllus, C. lucanusianus	0	0	0.436	P3P2	0.094	P3P2	0.004
C. guanaiensis var guanaiensis CA, C. sp. 18009, C. spiralis, C. lucanusianus	0.001	0	0.348	P3P2	0.112	P3P2	0.064
C. varzearum, C. erythrophyllus, C. sp. 18009, C. lucanusianus	0.368	0	1	P3P2	0	P3P2	0
C. varzearum, C. spiralis, C. sp. 18009, C. lucanusianus	0.16	0	0.999	P3P2	0	P2P3	0.422
C. sp. 18009, C. guanaiensis var guanaiensis CA, C. sp. nov. 19248, C. lucanusianus	0.003	0	0.007	P3P2	0.338	P3P2	0.144
C. erythrothyrsus, C. sp. nov. 19248, C. guanaiensis var guanaiensis CA, C. lucanusianus	0.189	0.097	0.916	P3P2	0.128	P3P2	0.484
C. varzearum, C. arabicus, C. sp. 18009, C. lucanusianus	0.04	0	0.719	P3P2	0.004	P2P3	0.306
C. guanaiensis var guanaiensis CA, C. sp. 18009, C. arabicus , C. lucanusianus	0	0.003	0.001	P2P3	0.688	P2P3	0.9

Table S2 continued from previous page

P1, P2, P3, O	P-value for ΔK_{23}	P-value for ΔK_{12}	P-value for ΔK_{13}	2xDIP direction	2xDIP p-value	3xDIP direction	3xDIP p-value
C. malortieanus, C. amazonicus, C. spicatus, C. lucanusianus	0	0	0.069	P3P2	0.468	P3P2	0.084
C. scaber SA, C. spicatus, C. amazonicus, C. lucanusianus	0.276	0	0.92	P3P2	0	P3P2	0

Table S3. For each colored cell in Figure 2, this table shows the corresponding f-branch metric, z-score, and lists any hybridization events from Table 1 and Figure 1 in the main text that are supported by the significant f-branch metric. F-branch metrics were calculated from admixture proportions associated with significant D-statistic tests (FWER < 0.001), using the gene-tree-based approach described in the Methods section. "Branch descendants" refers to the species or ancestral lineages on the y-axis, where multiple species separated by commas indicate that the branch subtending those species showed evidence of excess allele sharing. "Tip" refers to the species along the x-axis.

Branch descendants	Tip	F-branch	Z-score	Event
C_scaber_SA	C_woodsonii	0.252	11.677	event 3
C_spicatus	C_amazonicus	0.190	8.463	event 2
C_woodsonii	C_nitidus	0.099	3.855	
C_woodsonii	C_plicatus	0.104	5.388	
C_amazonicus	C_spicatus	0.210	5.750	event 2
C_leucanthus, C_guanaiensis_var_macrostrobilus, C_sp_19207, C_gibbosus, C_amazonicus_subsp_krukovii, C_acreanus_19245, C_acreanus_19253, C_guanaiensis_var_guanaiensis_SA, C_erythrophyllus, C_spiralis, C_arabicus, C_varzearum, C_guanaiensis_var_guanaiensis_CA, C_sp_18009, C_longebracteolatus, C_erythrocoryne, C_erythrothyrsus, C_sp_nov_19248, C_asplundii, C_zamoranus	C_spicatus	0.081	5.521	

Continued on next page

Table S3 – continued from previous page

Branch descendants	Tip	F-branch	Z-score	Event
C_woodsonii	C_pulverulentus	0.123	5.860	
C_barbatus, C_montanus	C_sp_nov_18049	0.338	6.566	
C_woodsonii	C_scaber_CA	0.113	4.121	
C_scaber_SA	C_scaber_CA	0.158	7.227	event 4
C_woodsonii	C_ricus	0.099	4.384	
C_amazonicus	C_leucanthus	0.167	6.559	event 1
C_spicatus	C_leucanthus	0.162	5.777	
C_malortieanus, C_amazonicus	C_guanaiensis_var_macrostrobilus	0.014	5.485	
C_amazonicus	C_guanaiensis_var_macrostrobilus	0.256	13.150	event 1
C_spicatus	C_guanaiensis_var_macrostrobilus	0.197	10.382	
C_amazonicus	C_sp_19207	0.208	10.566	event 1
C_spicatus	C_sp_19207	0.174	9.285	
C_amazonicus	C_gibbosus	0.227	12.136	event 1
C_spicatus	C_gibbosus	0.172	6.753	
C_amazonicus	C_guanaiensis_var_guanaiensis_CA	0.268	9.044	event 1
C_spicatus	C_guanaiensis_var_guanaiensis_CA	0.235	8.380	
C_amazonicus	C_sp_18009	0.277	17.087	event 1
C_spicatus	C_sp_18009	0.237	8.423	
C_villosissimus	C_sp_18009	0.031	4.515	
C_amazonicus	C_amazonicus_subsp_krukovii	0.326	11.352	event 1
C_spicatus	C_amazonicus_subsp_krukovii	0.230	9.156	

Continued on next page

Table S3 – continued from previous page

Branch descendants	Tip	F-branch	Z-score	Event
C_amazonicus	C_acreanus_19245	0.365	10.114	event 1
C_spicatus	C_acreanus_19245	0.229	7.914	
C_amazonicus	C_acreanus_19253	0.301	5.631	event 1
C_spicatus	C_acreanus_19253	0.261	10.380	
C_amazonicus	C_guanaiensis_var_guanaiensis_SA	0.289	13.286	event 1
C_spicatus	C_guanaiensis_var_guanaiensis_SA	0.263	15.838	
C_amazonicus	C_erythrophyllus	0.305	9.917	event 1
C_spicatus	C_erythrophyllus	0.192	11.862	
C_sp_18009	C_erythrophyllus	0.144	6.475	event 6
C_amazonicus	C_spiralis	0.288	25.863	event 1
C_spicatus	C_spiralis	0.223	8.380	
C_amazonicus	C_arabicus	0.334	14.060	event 1
C_spicatus	C_arabicus	0.237	11.977	
C_amazonicus	C_varzearum	0.295	18.352	event 1
C_spicatus	C_varzearum	0.243	10.806	
C_amazonicus	C_longebracteolatus	0.284	15.702	event 1
C_spicatus	C_longebracteolatus	0.237	12.584	
C_amazonicus	C_erythrocoryne	0.261	16.583	event 1
C_spicatus	C_erythrocoryne	0.239	15.236	
C_malortieanus, C_amazonicus	C_erythrothyrsus	0.025	5.115	
C_amazonicus	C_erythrothyrsus	0.268	11.732	event 1

Continued on next page

Table S3 – continued from previous page

**Branch descendants**	**Tip**	**F-branch**	**Z-score**	**Event**
C_spicatus	C_erythrothyrsus	0.230	12.571	
C_amazonicus	C_sp_nov_19248	0.261	8.323	event 1
C_spicatus	C_sp_nov_19248	0.222	10.475	
C_guanaiensis_var_guanaiensis_CA	C_sp_nov_19248	0.119	5.602	event 8
C_malortieanus, C_amazonicus	C_asplundii	0.010	5.836	
C_amazonicus	C_asplundii	0.284	12.938	event 1
C_spicatus	C_asplundii	0.238	10.520	
C_villosissimus	C_asplundii	0.038	5.393	
C_amazonicus	C_zamoranus	0.277	18.203	event 1
C_spicatus	C_zamoranus	0.242	11.783	

Notes S1.

Site pattern frequencies in significant vs. non-significant *D*-statistic tests

The *D*-statistic assesses gene flow between species by comparing the site pattern frequencies exhibited by four taxa: P1, P2, P3, and an outgroup. In this context, BBAA represents P1 and P2 sharing the derived allele, ABBA represents P2 and P3 sharing the derived allele, and BABA represents P1 and P3 sharing the derived allele. Assuming the relationships among taxa are (((P1, P2), P3), P4), BBAA is consistent with the tree, while ABBA and BABA arise from incomplete lineage sorting or introgression between P1 or P2 and P3. Incomplete lineage sorting is expected to increase the number of ABBA and BABA site patterns (nABBA and nBABA) equally, while gene flow is expected to preferentially increase nABBA or nBABA, leading to a positive *D*-statistic.

We conducted our *D*-statistic analyses using `Dsuite Dtrios` [Malinsky *et al.*, 2021]. Instead of allowing `Dtrios` to infer the species tree based on the most common site pattern, we arranged the taxa (P1, P2, P3) according to a robust maximum likelihood Costus phylogeny (Vargas et al., 2020). P1 and P2 are always ordered so that nABBA $>$ nBABA. Of the 24,804 tests, 2,374 (9.6%) were significant, and 22,430 (90.4%) were not significant at an alpha level of 0.001 (Holm-Bonferroni adjusted). In 93% of non-significant tests, nBBAA $>$ nABBA, i.e., the most frequent site pattern was consistent with the species tree. However, in 85% of significant tests, nABBA $>$ nBBAA, i.e., the most frequent site pattern either reflects a history of introgression or the species tree is incorrect.

Although we cannot entirely rule out errors in the species tree, we contend that the abundance of significant tests with nABBA $>$ nBBAA is largely due to gene flow between P2 and P3. In the paper describing the `Dsuite` software package [Malinsky *et al.*, 2021], the authors analyzed a small simulated data set and found that the true sister species did not share the derived allele in 39 cases (3.4% of trios), which they attributed to gene flow. In our analysis, significant tests where nABBA $>$ nBBAA exhibit significantly higher introgression proportions than those with nBBAA $>$ nABBA (95% CI for the between-group difference in mean introgression proportions $= 0.18 - 0.2$), suggesting that higher levels of gene flow are more likely to cause this pattern.

Notes S2.

DIP analyses to detect and polarize asymmetric introgression

The 3×DIP analysis of introgression between *C. amazonicus* and *C. spicatus* was highly significant ($P < 0.05$) when South American *C. scaber*, *C. spicatus*, and *C. amazonicus* were chosen for P1, P2, and P3, and indicated introgression from *C. amazonicus* into *C. spicatus*, however, the analysis was not significant ($P = 0.084$) when *C. malortieanus*, *C. amazonicus*, and *C. spicatus* were chosen for P1, P2, and P3. To polarize the direction of introgression between *C. amazonicus* and the ancestor of the Amazonian clade, we conducted 3×DIP analyses with *C. malortieanus* and *C. amazonicus* as P1 and P2 and each member of the Amazonian clade as P3, resulting in 20 analyses. Half of the 3×DIP analyses were significant after Holm-Bonferroni adjustment and supported directional introgression from the ancestor of the Amazonian clade into *C. amazonicus*. The 3×DIP analysis of introgression between *C. flammulus* and *C. montanus* was significant ($P = 0.002$) and indicated asymmetric introgression from *C. montanus* into *C. flammulus* when *C. pulverulentus*, *C. flammulus*, and *C. montanus* were chosen for P1, P2, and P3; however, the 3×DIP analysis was not significant when *C. barbatus*, *C. montanus*, and *C. flammulus* were chosen for P1, P2, and P3 ($P = 0.278$). The 3×DIP analysis of introgression between *C. sp.* 18009 and the ancestor of *C. erythrophyllus* and *C. spiralis* was significant ($P = 0.004$) when Central American *C. guanaiensis* var. *guanaiensis*, *C. sp.* 18009, and *C. erythrophyllus* were chosen for P1, P2, and P3, but not significant ($P = 0.064$) when *C. spiralis* replaced *C. erythrophyllus* as P3, and thus the prevailing direction of introgression could not be resolved. Similar procedures were conducted to polarize introgression between Central American *C. guanaiensis* var. *guanaiensis* and *C. sp.* nov. 19248, *C. arabicus* and *C. sp.* 18009, and South American *C. scaber* and *C. woodsonii*, but none of the 3×DIP analyses were significant, suggesting bidirectional introgression between these three pairs of taxa. Finally, to polarize the direction of introgression between the South American *C. scaber* accession and the ancestor of the *C. pulverulentus* clade (containing *C. nitidus*, *C. plicatus*, *C. pulverulentus*, *C. flammulus*, *C. ricus*, and Central American *C. scaber*), we conducted 3×DIP analyses with *C. spicatus* and South American *C. scaber* as P1 and P2 and each member of the *C. pulverulentus* clade as P3, resulting in six analyses. None of these analyses were significant, suggesting bidirectional introgression between South American *C. scaber* and the ancestor of the *C. pulverulentus* clade.

Notes S3.

Pseudo-likelihood phylogenetic network inference with PhyloNet

Phylogenetic networks were inferred with the maximum pseudo-likelihood inference method included in `PhyloNet` [Than *et al.*, 2008, Yu & Nakhleh, 2015]. To ease computational intensity, we split the samples into four taxon subsets and used a tree-based augmentation approach whereby a previously inferred maximum-likelihood *Costus* phylogeny [Vargas *et al.*, 2020] was fixed as the backbone tree and augmented into a network using the *-fs* option with the `InferNetwork_MPL` method. To assess the influence of using different bootstrap thresholds when contracting poorly supported gene tree edges, we independently analyzed three bootstrap support thresholds (70, 80, and 90%) per taxon subset and compared the results of each. For each taxon subset and bootstrap threshold, a slope heuristic approach [Solís-Lemus & Ané, 2016] was used to identify the best maximum number of reticulations (0–3).

The first taxon subset ("clade 1") is a clade of 13 predominantly Central American taxa that diverged early in the history of *Costus*. For this clade, the addition of a reticulation edge was only favored with the data set generated using the 70% bootstrap threshold (Fig. S8). For this data set, a reticulation edge with an inheritance probability of 0.417 was inferred from the sister species *C. amazonicus* and *C. malortieanus* into sister species *C. spicatus* and South American *C. scaber* (Fig. S9A). This event is consistent with introgression from *C. amazonicus* into *C. spicatus* that was inferred with the RT test analysis (Fig. 1) and the f-branch analysis (Fig. 2), and is similar to the reticulation edge inferred with `SNaQ` (Fig. 3). However, both `PhyloNet` (γ = 0.417) and `SNaQ` (γ = 0.452) inferred much higher inheritance probabilities for this event than the RT test (γ = 0.22) and f-branch analyses (γ = 0.15).

The second taxon subset ("grade 2") contains a grade of 20 taxa that connects clades one and three. For all bootstrap thresholds, the addition of reticulation edges to the backbone tree did not substantially improve the pseudo-likelihood according to a slope heuristic approach (Fig. S8). A lack of introgression in this grade is consistent with absent and weak signatures of introgression inferred, respectively, with RT test and f-branch analyses for this subset of taxa (Fig. 2, 3).

The third taxon subset ("clade 3") contains a young clade of twenty predominantly-Amazonian taxa. For the data sets generated using bootstrap thresholds of 70 and 90%, the addition of reticulation edges to the backbone tree did not substantially improve the pseudo-likelihoods according to a slope heuristic (Fig. S8). However, when using a bootstrap threshold of 80%, the addition of two reticulation edges substantially improved the pseudo-likelihood, although both reticulation edges have low inheritance probabilities. First, introgression with an inheritance probability of 0.0316 was inferred from sister species *C. sp.* 18009 and Central American *C. guanaiensis* var. *guanaiensis* into the clade containing *C. sp. nov.* 19248, *C.*

asplundii, *C. erythrothyrsus*, *C. zamoranus*, *C. erythrocoryne*, and *C. longebracteolatus* (Fig. S11B). This reticulation was associated with a large-scale change in tree topology, with the recipient clade moving to a more ancestral position in the tree, and the sister species *C. sp.* 18009 and Central American *C. guanaiensis* var. *guanaiensis* placed sister to the clade containing *C. acreanus*, *C. amazonicus* var. *krukovii*, South American *c. guanaiensis* var. *guanaiensis*, *C spiralis*, *C. erythrophyllus*, *C. arabicus*, and *C. varzearum.* This affinity of sister species *C. sp.* 18009 and Central American *C. guanaiensis* var. *guanaiensis* with the clade containing *C. erythrophyllus* and *C. spiralis* is consistent with introgression between *C. sp.* 18009 and the ancestral lineage subtending sister species *C. erythrophyllus* and *C. spiralis*, which was inferred with the RT test analysis. Second, introgression with an inheritance probability of 0.0233 was inferred from *C. sp. nov.* 19248 into *C. erythrothyrsus* (Fig. S11B). Since *C. sp. nov.* 19248 and *C. erythrothyrsus* were resolved as sister taxa in the maximum likelihood phylogeny of Vargas *et al.* [2020], this event may reflect topological uncertainty caused by gene tree discordance rather than true introgression.

The fourth taxon subset ("subset 4") contains 20 taxa that span the deepest divergences in the American *Costus* phylogeny. The addition of two reticulation edges using a bootstrap threshold of 70% and one reticulation edge using a bootstrap threshold of 80% provided substantial increases in pseudo-likelihood, but no improvement was observed with the addition of reticulation edges using a 90% bootstrap threshold (Fig. S8). Using a bootstrap threshold of 70%, one reticulation edge with an inheritance probability of 0.211 was inferred from *C. spicatus* into the ancestor of sister species *C. chartaceus* and *C. vargasii* (Fig. S12A). This event was not resolved with other analyses. The second reticulation edge with an inheritance probability of 0.464 was inferred from the ancestor of *C. ricus* and *C. pulverulentus* into *C. barbatus* (Fig. S12A). This event is consistent with f-branch results, which provided evidence for introgression between the ancestor of the sister species, *C. barbatus* and *C. montanus*, and the ancestor of the clade containing *C. pulverulentus*, *C. flammulus*, Central American *C. scaber*, and *C. ricus* (Fig. 2). Using a bootstrap threshold of 80%, one reticulation edge with an inheritance probability of 0.263 was inferred from *C. gibbosus* into a subclade of clade three containing South American *C. guanaiensis* var. *guanaiensis*, *C. varzearum*, *C. longebracteolatus*, *C. erythrothyrsus*, and *C. asplundii* as the recipient (Fig. S12B). The addition of this reticulation edge was accompanied by a significant change to the backbone topology, namely, the recipient subclade of clade three was placed ancestrally to the clade containing representative samples from clade one (*C. spicatus*, *C. pulverulentus*, *C. ricus*, *C. nitidus*, *C. malortieanus*, and *C. amazonicus*). A potential explanation for this affinity between the clade three subclade and clade one is introgression from the ancestor of the Amazonian clade into *C. amazonicus*, as was inferred by the RT test and f-branch analyses.

References

Forsythe ES, Sloan DB , Beilstein MA. 2020. Divergence-Based Introgression Polarization. *Genome Biology and Evolution*, **12**: 463–478.

Malinsky M, Matschiner M , Svardal H. 2021. Dsuite - Fast D-statistics and related admixture evidence from VCF files. *Molecular Ecology Resources*, **21**: 584–595.

Solís-Lemus C , Ané C. 2016. Inferring Phylogenetic Networks with Maximum Pseudolikelihood under Incomplete Lineage Sorting. *PLOS Genetics*, **12**: e1005896.

Than C, Ruths D , Nakhleh L. 2008. PhyloNet: a software package for analyzing and reconstructing reticulate evolutionary relationships. *BMC Bioinformatics*, **9**: 322.

Vargas OM, Goldston B, Grossenbacher DL , Kay KM. 2020. Patterns of speciation are similar across mountainous and lowland regions for a Neotropical plant radiation (Costaceae: *Costus*). *Evolution*, **74**: 2644–2661.

Yu Y , Nakhleh L. 2015. A maximum pseudo-likelihood approach for phylogenetic networks. *BMC Genomics*, **16**: S10.
